# Supplementary material for: Impact of international travel and diarrhea on gut microbiome and resistome dynamics
Source: Nat Commun. 2022 Dec 5;13:7485. doi: 10.1038/s41467-022-34862-w (PMC9722912; doi:10.1038/s41467-022-34862-w)
Supplement: Supplementary file 1 — Supplementary Information [file 41467_2022_34862_MOESM1_ESM.pdf]

Supplementary Information

Impact of international travel and diarrhea on gut microbiome and resistome dynamics

Manish Boolchandani[1,2], Kevin S. Blake[1,2], Drake H. Tilley[3], Miguel M. Cabada[4,5], Drew J. Schwartz[1,6,7,8,9], Sanket Patel[1,2], Maria Luisa Morales[5], Rina Meza[3], Giselle Soto[3], Sandra D. Isidean[10,11], Chad K. Porter[10], Mark P. Simons[3,10*], Gautam Dantas[1,2,7,12*]

[1] The Edison Family Center for Genome Sciences and Systems Biology, Washington University School of Medicine, St. Louis, MO, USA

[2] Department of Pathology and Immunology, Washington University School of Medicine, St. Louis, MO, USA

[3] Naval Medical Research Unit No. 6, Callao, Lima, Peru

[4] Department of Internal Medicine, Division of Infectious Diseases, University of Texas Medical Branch, Galveston, TX, USA

[5] Cusco Branch – Tropical Medicine Institute, Universidad Peruana Cayetano Heredia, Lima, Peru

[6] Department of Pediatrics, Division of Infectious Diseases, Washington University School of Medicine, St. Louis, MO, USA

[7] Department of Molecular Microbiology, Washington University School of Medicine, St. Louis, MO, USA

[8] Department of Obstetrics and Gynecology, Washington University in St. Louis, St. Louis, MO, USA

[9] Center for Women's Infectious Diseases Research, Washington University in St. Louis, St. Louis, MO, USA

[10] Naval Medical Research Center, Silver Spring, MD, USA

[11] Henry M. Jackson Foundation for the Advancement of Military Medicine, Inc., Bethesda, MD, USA

[12] Department of Biomedical Engineering, Washington University in St. Louis, St. Louis, MO, USA

Supplementary Figures

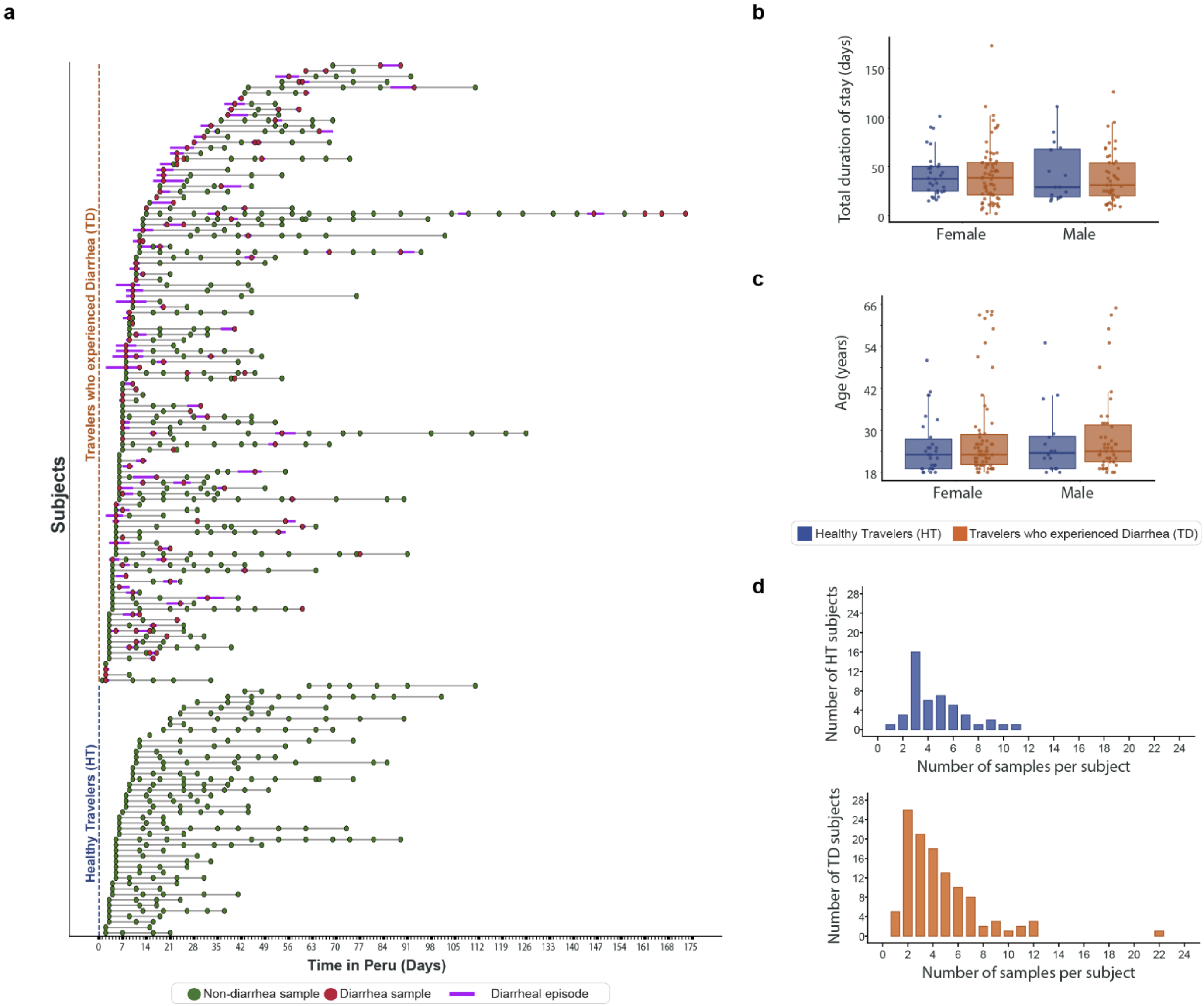

Supplementary Fig. 1 a) Timeline of sample collection and diarrheal episode among travelers with diarrhea (TD, top) and healthy travelers (HT, bottom). The non-diarrhea samples are represented in green and diarrhea samples are indicated in red with diarrhea duration indicated in pink color bars. **b-d)** Distribution of duration of stay, age, and number of processed samples per subject in HT and TD group (boxes show medians/quartiles; error bars extend to the most extreme values within 1.5 interquartile ranges).

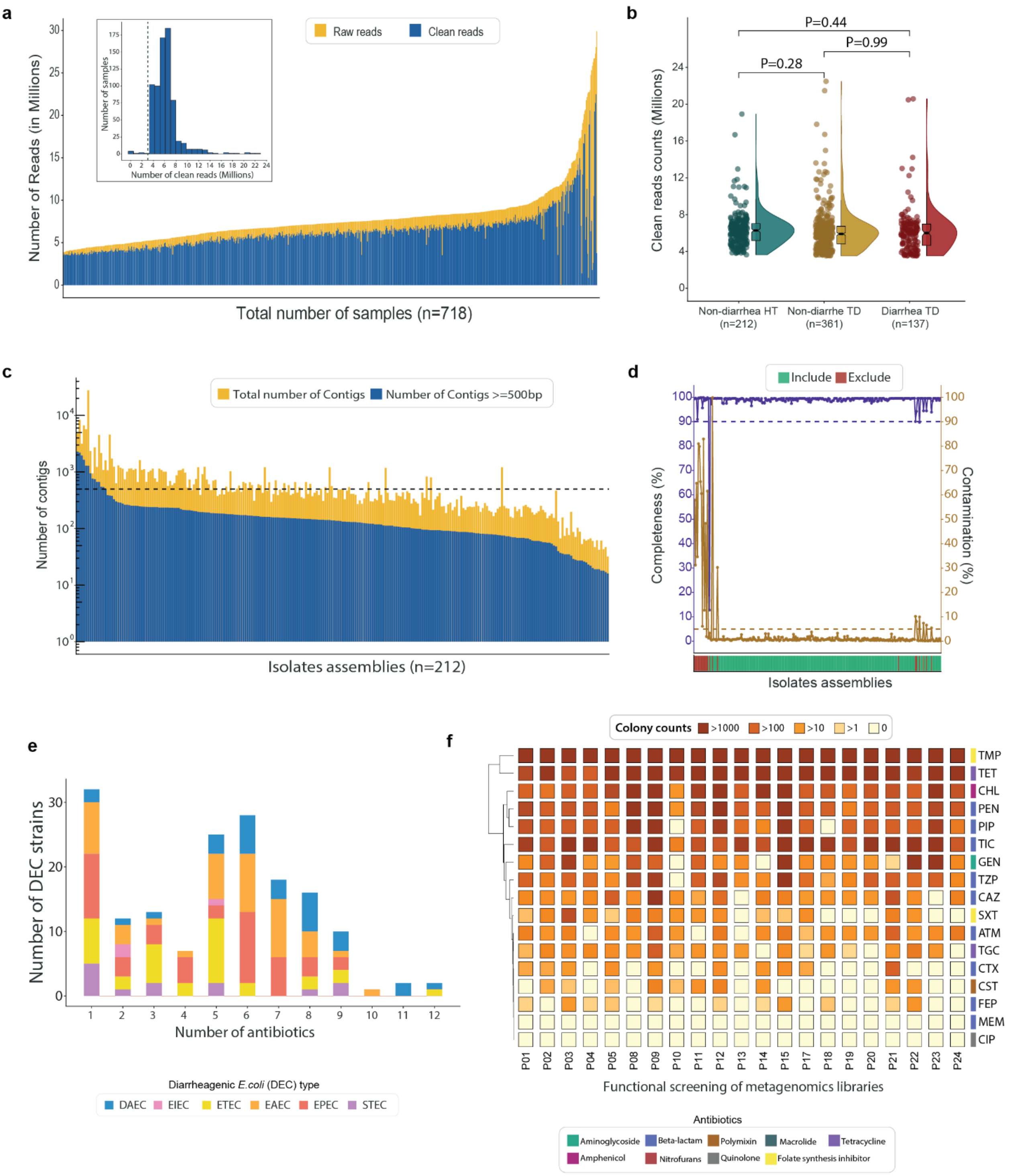

Supplementary Fig. 2: Summary of shortgun metagenomics, isolates sequencing and functional metagenomics. a) Distribution of unprocessed (yellow) and quality-filtered trimmed reads (blue) in metagenomics samples (n=718). (inset) Distribution of cleaned reads (in millions) where samples with less than 3M cleaned reads were discarded. **b)** Pairwise comparison of cleaned read counts across sample types from HT and TD Subjects. Two-sided Student's t-test was used to check the differences between groups. Boxes show medians/quartiles; error bars extend to the most extreme values within 1.5 interquartile ranges. **c)** Total number of contigs in each isolate assembly (highlighted in yellow) and the filtered contigs with length greater than 500bp (highlighted in blue). **d)** Line plot depicting the completeness and contamination of isolate assemblies. Isolate assemblies with > 90% completeness and < 5% contamination were included in the analysis (represented by green bar on x-axis) while the remaining were excluded (red bar on the x-axis). **e)** Distribution of number of antibiotics towards which diarrheagenic E. coli (DEC) isolates were phenotypically resistant. f) Heatmap of the number of colonies observed during screening of functional metagenomics libraries (n=21) against 17 antibiotics.

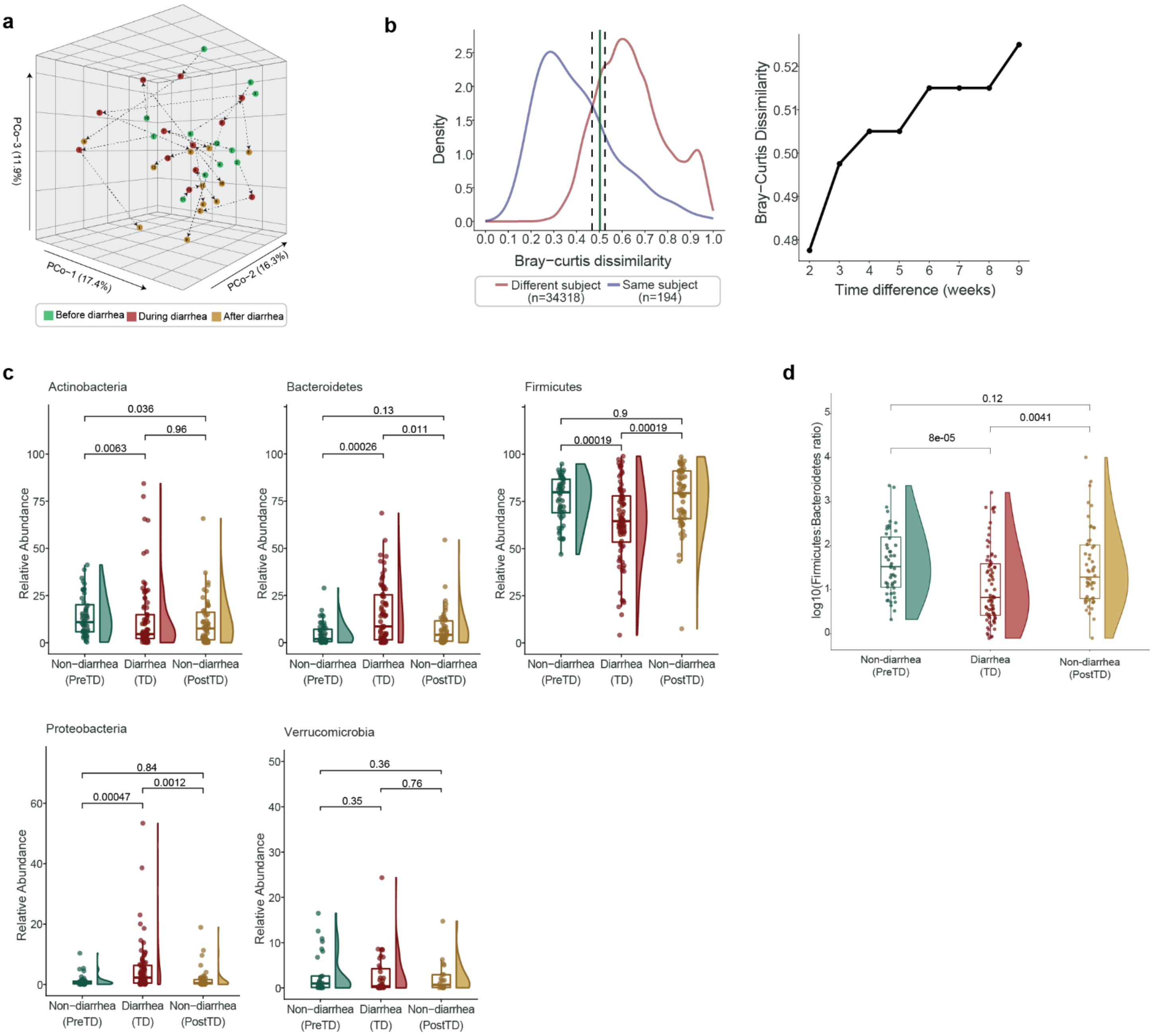

Supplementary Fig. 3: a) Principal Component Analysis (PCA) of subject-matched samples collected before, during, and after diarrheal episodes. Dotted lines connect samples from the same subject. **b)** Distribution of Bray-Curtis dissimilarity between consecutive samples from the same subject (represented by blue curve) versus those from different individuals (represented by red curve), with “microbiome shift events” defined as the Bray-Curtis threshold > 0.52 (represented by green line). **c)** Relative abundance of five major phyla in before, during, and after diarrhea samples (two-sided Wilcoxon test, n = 171 biologically independent samples). **d)** Distribution of log10 (Firmicutes:Bacteroidetes ratio) in before, during, and after diarrhea samples (two-sided Wilcoxon test, n = 171 biologically independent samples). Boxes show medians/quartiles; error bars extend to the most extreme values within 1.5 interquartile ranges.

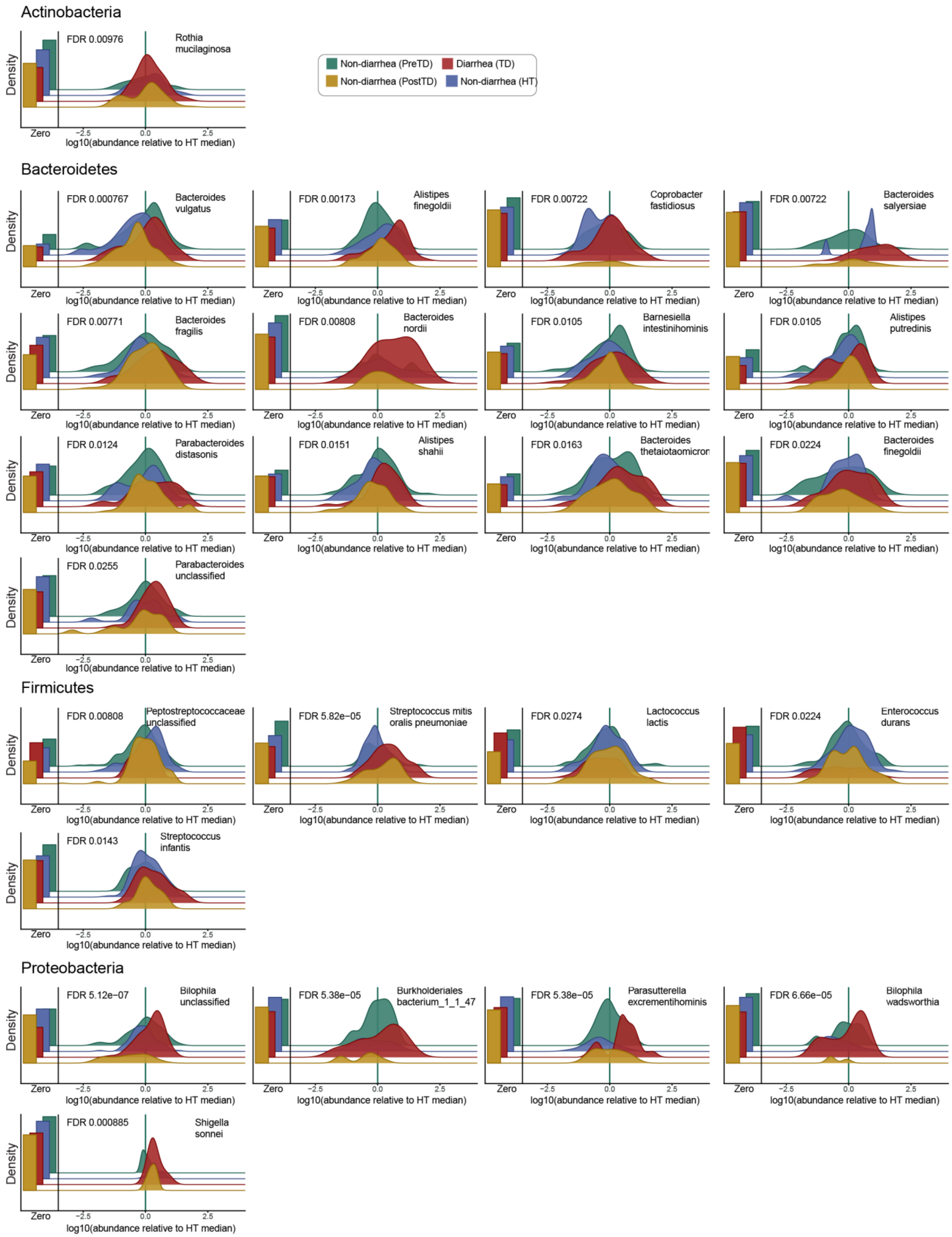

Supplementary Fig.4: Relative abundance distribution of differentially abundant species belonging to four phyla, normalized by the median relative abundance of non-diarrheal HT samples. Left barplot, fraction of samples below detection limit.

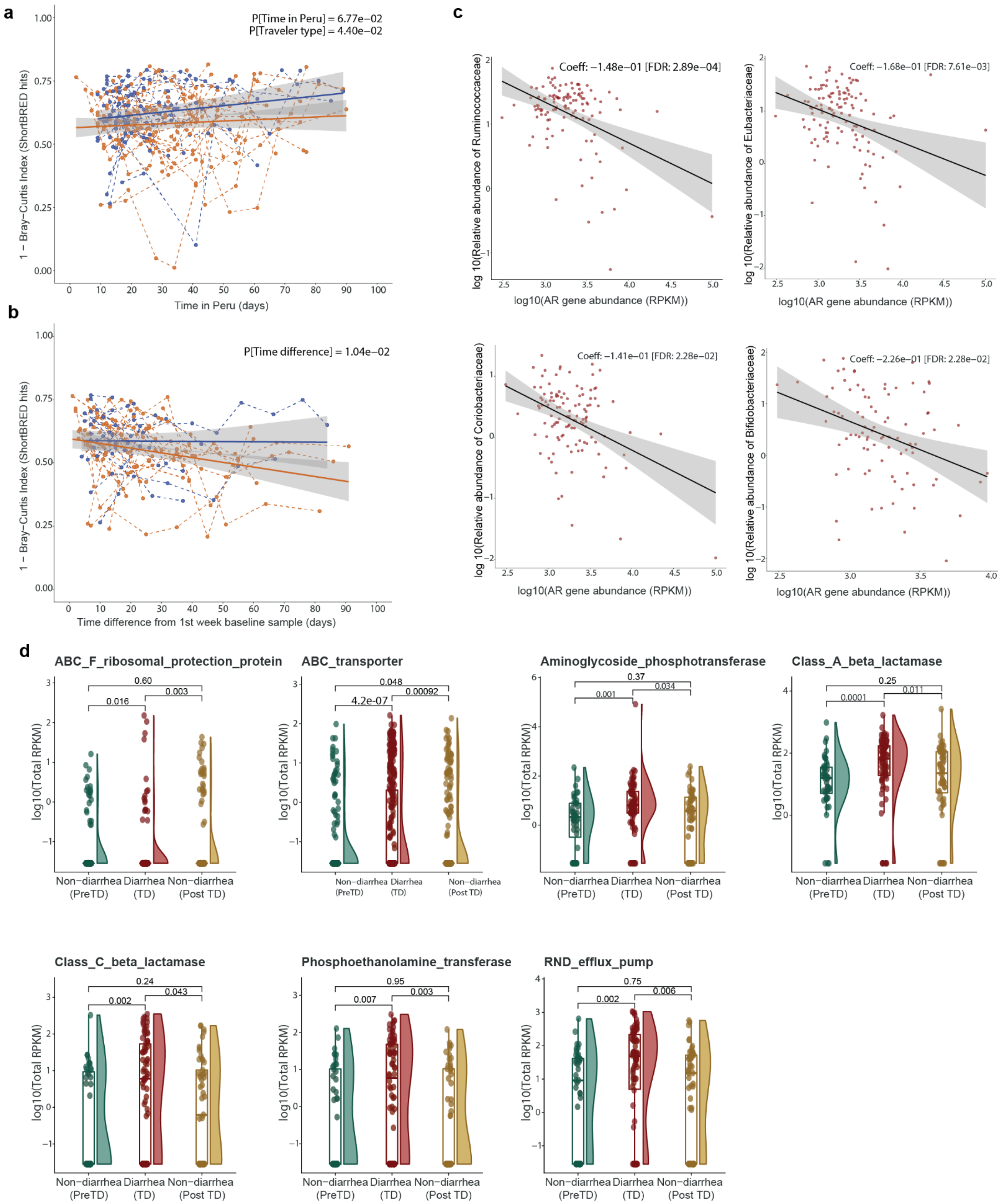

a
P[Time in Peru] = 6.77e−02
P[Traveler type] = 4.40e−02
1 − Bray−Curtis Index (ShortBRED hits)
Time in Peru (days)
b
P[Time difference] = 1.04e−02
1 − Bray−Curtis Index (ShortBRED hits)
Time difference from 1st week baseline sample (days)
c
Coeff: −1.48e−01 [FDR: 2.89e−04]
log 10(Relative abundance of Ruminococcaceae)
log10(AR gene abundance (RPKM))
Coeff: −1.68e−01 [FDR: 7.61e−03]
log 10(Relative abundance of Eubacteriaceae)
log10(AR gene abundance (RPKM))
Coeff: −1.41e−01 [FDR: 2.28e−02]
log 10(Relative abundance of Coriobacteriaceae)
log10(AR gene abundance (RPKM))
Coeff: −2.26e−01 [FDR: 2.28e−02]
log 10(Relative abundance of Bifidobacteriaceae)
log10(AR gene abundance (RPKM))
d
ABC_F_ribosomal_protection_protein
0.60
0.016
0.003
ABC_transporter
0.048
4.2e-07
0.00092
Aminoglycoside_phosphotransferase
0.37
0.001
0.034
Class_A_beta_lactamase
0.25
0.0001
0.011
Class_C_beta_lactamase
0.24
0.002
0.043
Phosphoethanolamine_transferase
0.95
0.007
0.003
RND_efflux_pump
0.75
0.002
0.006
log10(Total RPKM)
Non−diarrhea (PreTD)
Diarrhea (TD)
Non−diarrhea (Post TD)

Supplementary Fig. 5: a) 1-Bray-Curtis dissimilarities of ARG compositions between consecutive samples from each subject, plotted throughout the length of their time in Peru with dotted lines connecting samples from the same subject. Points are individual fecal samples, colored by sample type (i.e diarrhea or non-diarrhea). Solid lines show the best fit from different traveler types: HT (blue) and TD (orange) (n=290, LMM, P > 0.05) and the gray shading represents the 95% confidence interval (CI). **b)** Bray-Curtis dissimilarities between each subjects' samples and their 1st-week baseline sample. Points are individual fecal samples, colored by sample type (i.e. diarrhea or non-diarrhea), with dotted lines connecting samples from the same subject. Solid lines shows best fit with 95% CI of samples from different traveler types: HT (blue) and TD (orange) (n=189, LMM P[Time difference] < 0.05). **c)** Increasing ARG abundance was associated with a decrease in microbial diversity (at the species level) of Ruminococcaceae, Eubacteriaceae, Coriabacteriaceae, and Bifidobacteriaceae. The significant associations were detected by MaAsLin2 where other metadata variables (age, sex, sample type, region, length of stay and antibiotics usage) were used as fixed effects. Solid lines shows best fit with gray shading representing 95% CI. The significant associations were corrected for multiple hypothesis testing using Benjamini-Hochberg method. **d)** ARG families with significant change in cumulative abundance (RPKM in log10 scale) during diarrheal episodes. Two-sided Wilcoxon test was used to check the differences between the groups (n=171 biologically independent samples). P-values are multiple hypothesis test corrected using Benjamini–Hochberg (FDR) method. Boxes show medians/quartiles; error bars extend to the most extreme values within 1.5 interquartile ranges.

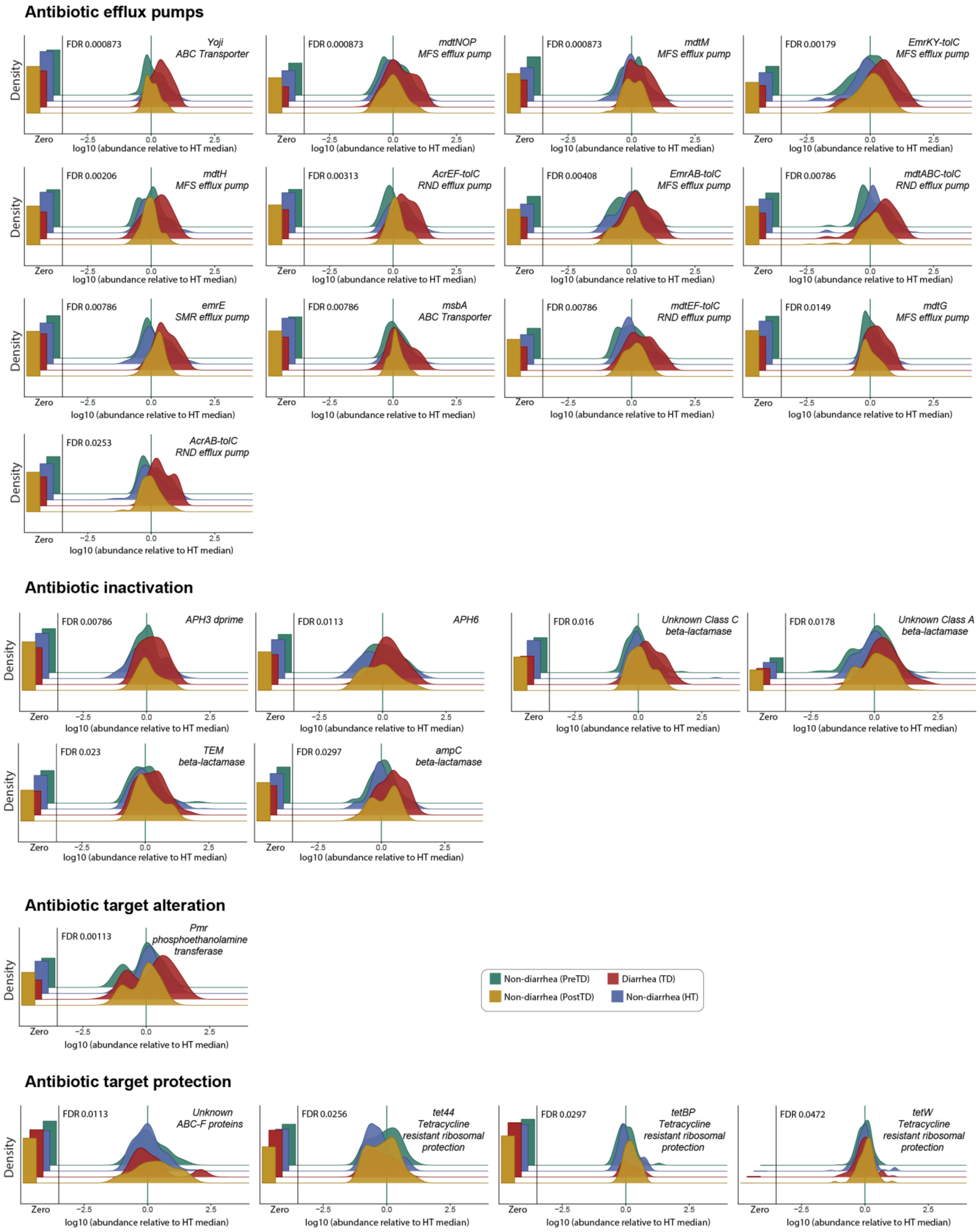

Supplementary Fig. 6: Relative abundance distribution of differentially abundant ARGs belonging to 4 resistance mechanisms, normalized by the median relative abundance of non-diarrheal HT samples. Left barplot, fraction of samples below detection limit.

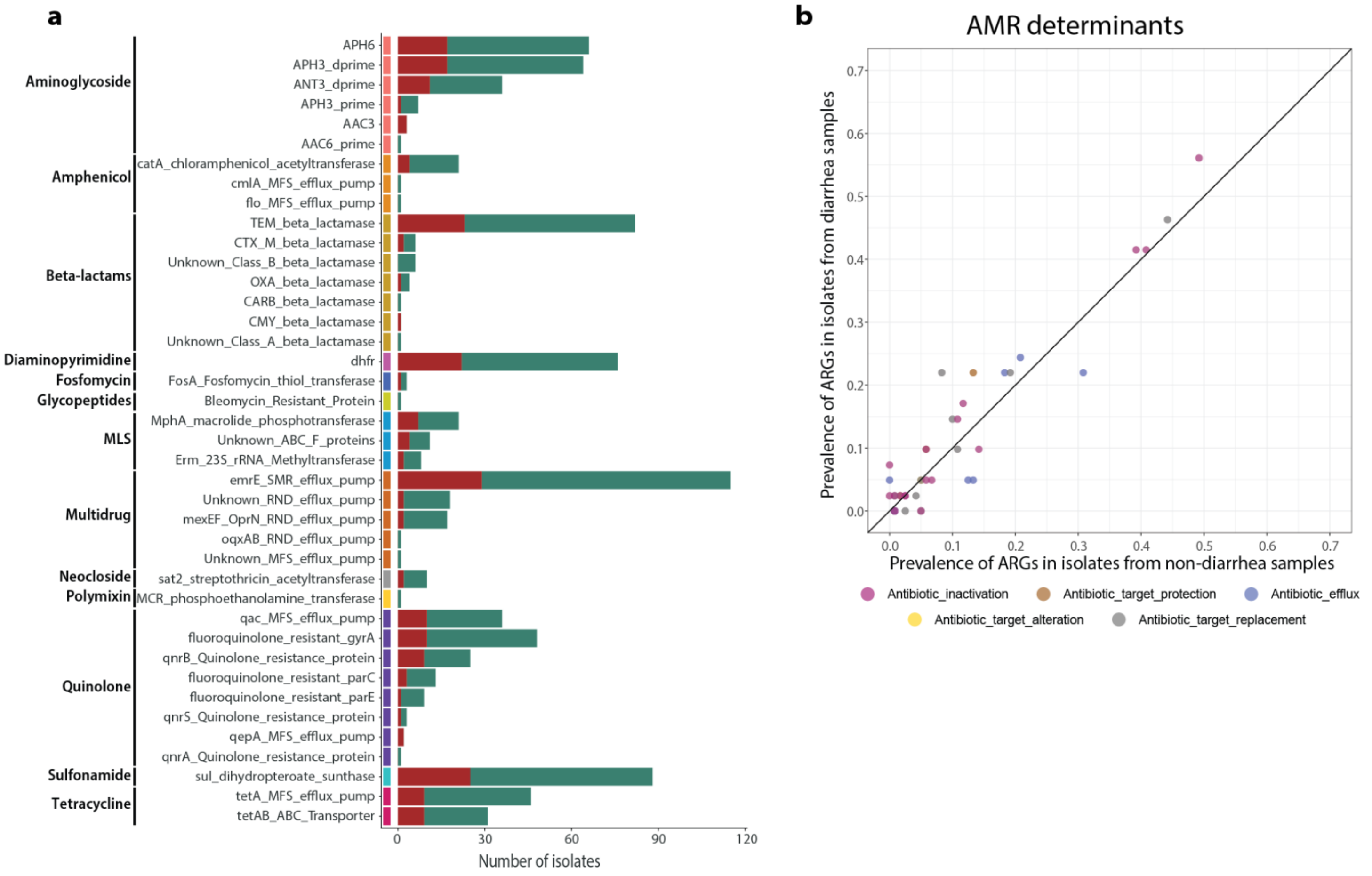

Supplementary Fig. 7: a) The frequency of antibiotic resistance gene families identified in the genomes of DEC strains isolated from non-diarrheal (green) and diarrheal (red) sample types. **b)** Prevalence of antibiotic resistance genes in DEC isolates from diarrheal or non-diarrheal samples.

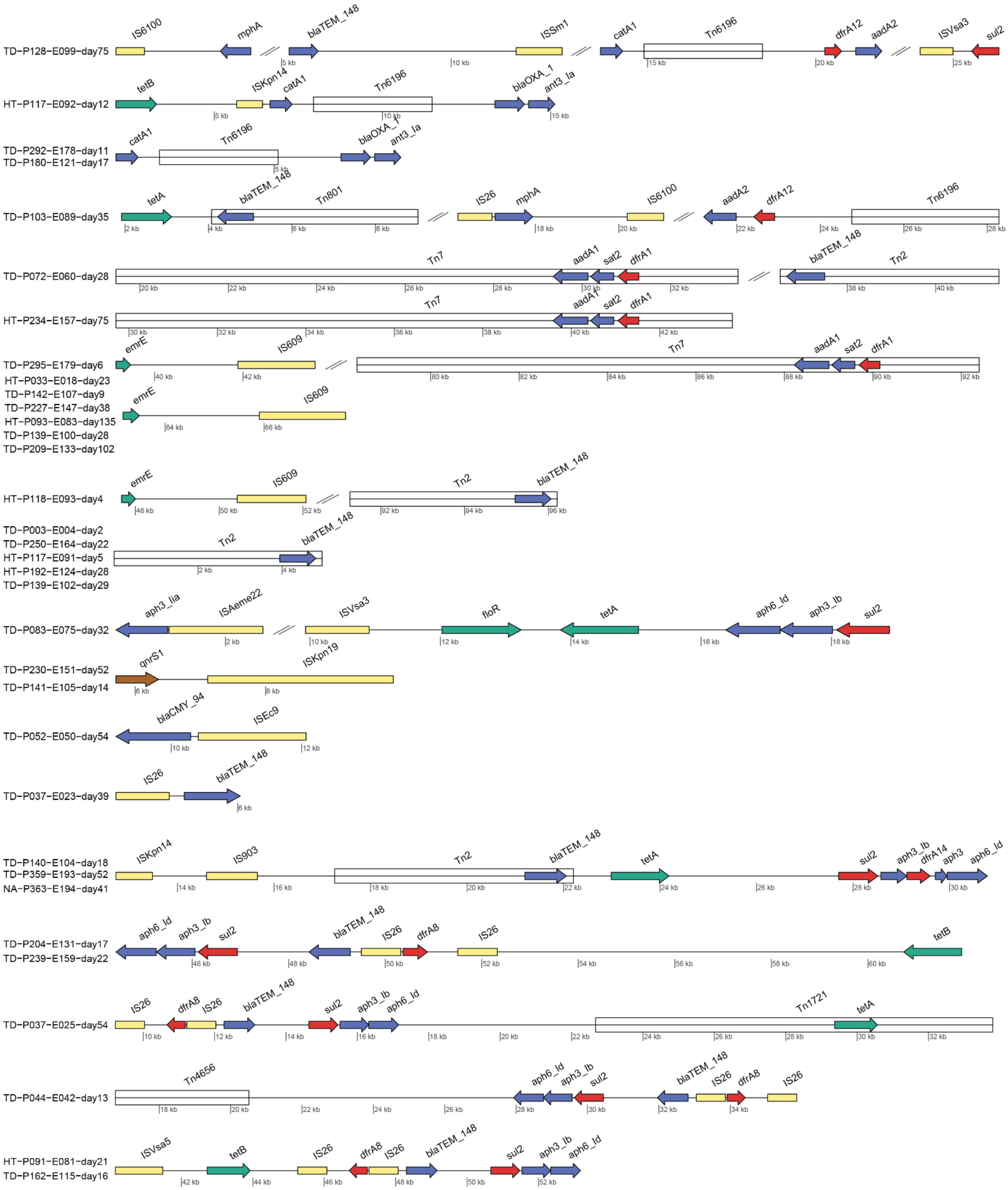
TD−P128−E099−day75
IS6100
mphA
blaTEM_148
ISSm1
catA1
Tn6196
dfrA12
aadA2
ISVsa3
sul2
HT−P117−E092−day12
tetB
ISKpn14
catA1
Tn6196
blaOXA_1
ant3_Ia
TD−P292−E178−day11
TD−P180−E121−day17
catA1
Tn6196
blaOXA_1
ant3_Ia
TD−P103−E089−day35
tetA
blaTEM_148
Tn801
IS26
mphA
IS6100
aadA2
dfrA12
Tn6196
TD−P072−E060−day28
Tn7
aadA1
sat2
dfrA1
blaTEM_148
Tn2
HT−P234−E157−day75
Tn7
aadA1
sat2
dfrA1
TD−P295−E179−day6
emrE
IS609
Tn7
aadA1
sat2
dfrA1
HT−P033−E018−day23
TD−P142−E107−day9
TD−P227−E147−day38
HT−P093−E083−day135
TD−P139−E100−day28
TD−P209−E133−day102
emrE
IS609
HT−P118−E093−day4
emrE
IS609
Tn2
blaTEM_148
TD−P003−E004−day2
TD−P250−E164−day22
HT−P117−E091−day5
HT−P192−E124−day28
TD−P139−E102−day29
Tn2
blaTEM_148
TD−P083−E075−day32
aph3_IIa
ISAeme22
ISVsa3
floR
tetA
aph6_Id
aph3_Ib
sul2
TD−P230−E151−day52
TD−P141−E105−day14
qnrS1
ISKpn19
TD−P052−E050−day54
blaCMY_94
ISEc9
TD−P037−E023−day39
IS26
blaTEM_148
TD−P140−E104−day18
TD−P359−E193−day52
NA−P363−E194−day41
ISKpn14
IS903
Tn2
blaTEM_148
tetA
sul2
aph3_Ib
dfrA14
aph3
aph6_Id
TD−P204−E131−day17
TD−P239−E159−day22
aph6_Id
aph3_Ib
sul2
blaTEM_148
IS26
dfrA8
IS26
tetB
TD−P037−E025−day54
IS26
dfrA8
IS26
blaTEM_148
sul2
aph3_Ib
aph6_Id
Tn1721
tetA
TD−P044−E042−day13
Tn4656
aph6_Id
aph3_Ib
sul2
blaTEM_148
IS26
dfrA8
IS26
HT−P091−E081−day21
TD−P162−E115−day16
ISVsa5
tetB
IS26
dfrA8
IS26
blaTEM_148
sul2
aph3_Ib
aph6_Id

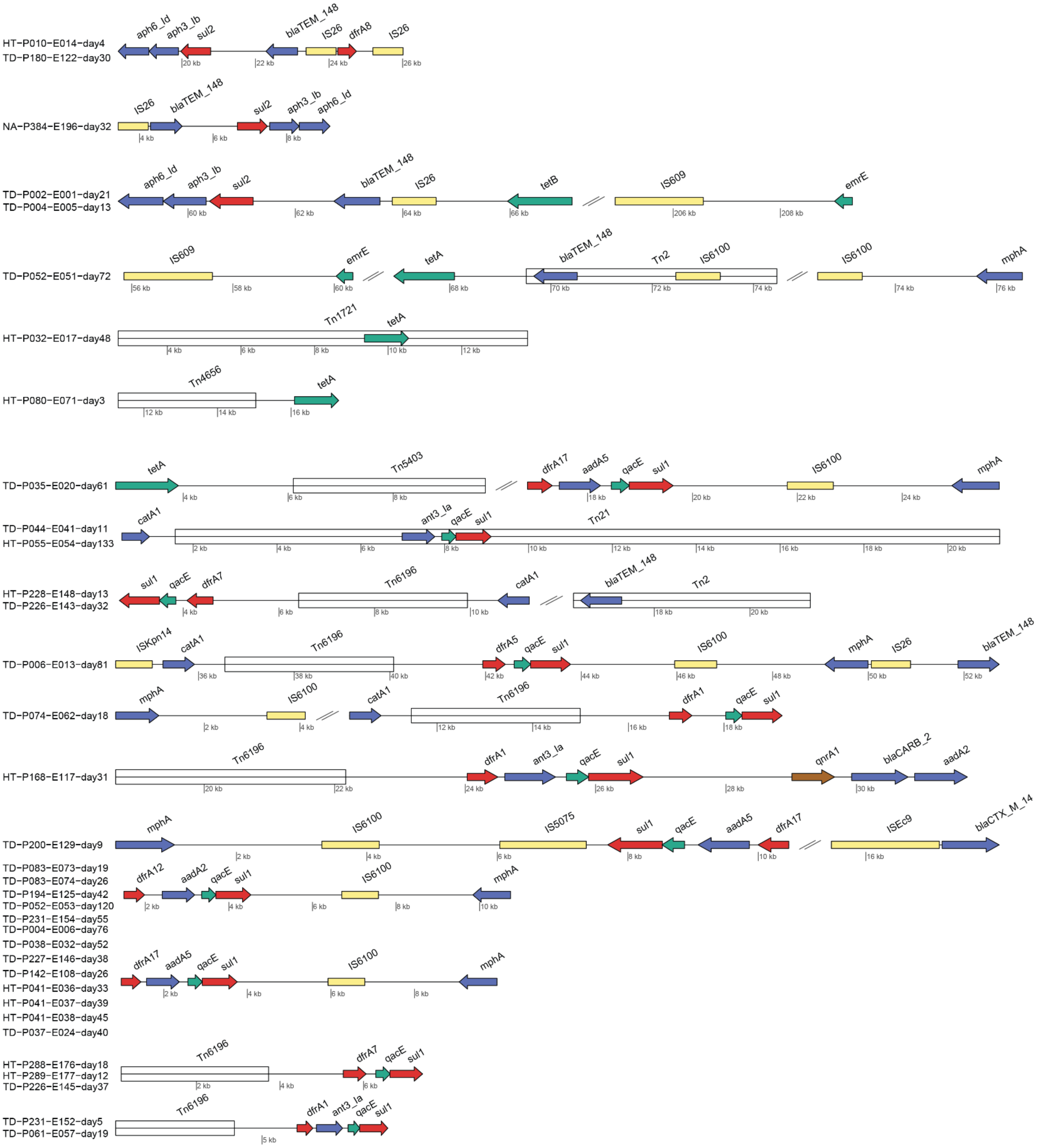

Supplementary Fig. 8: Examples of arrangement of ARGs and mobile elements identified on the same contigs from isolates.

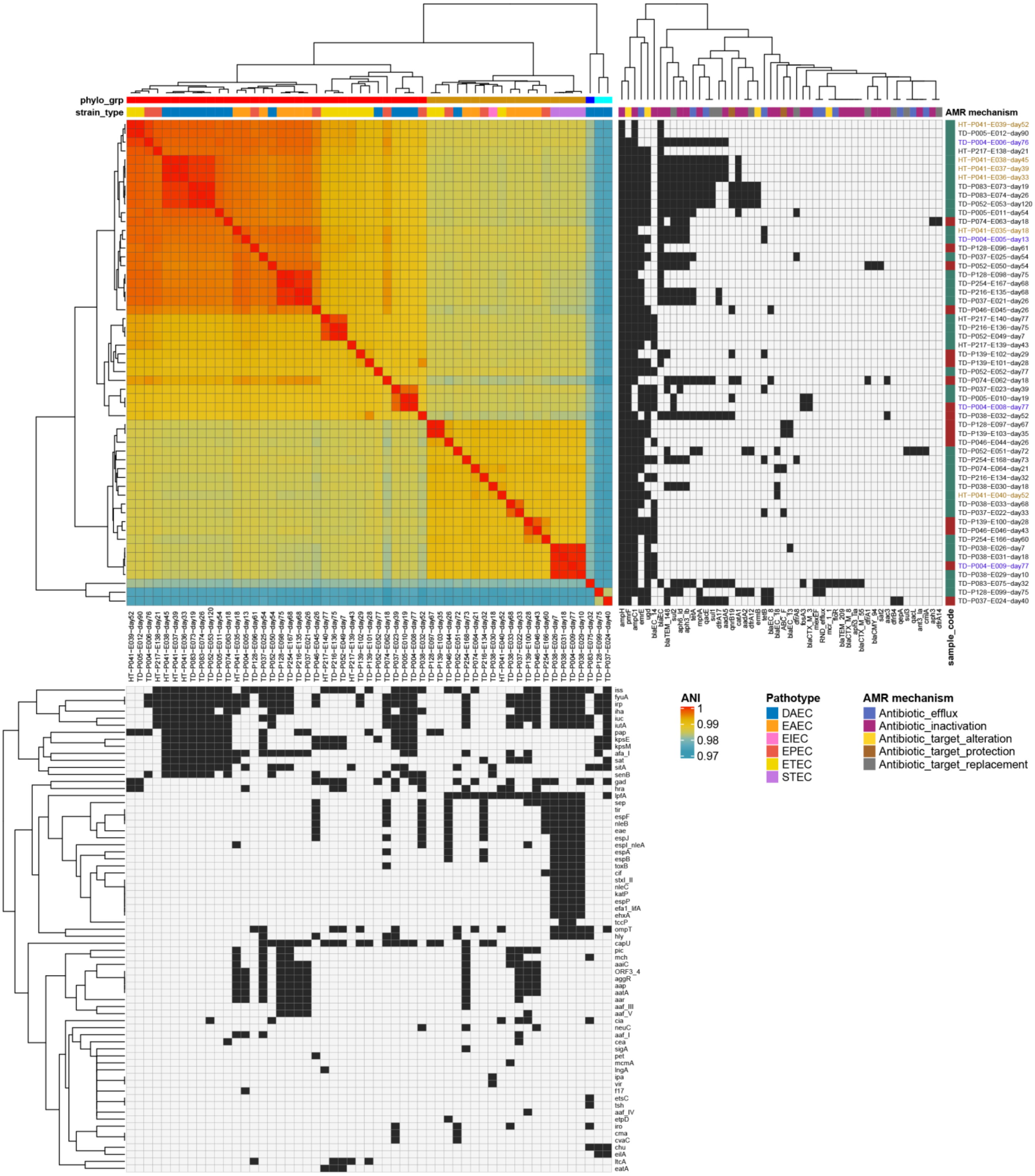

Supplementary Fig. 9: Relatedness of DEC isolates assessed by Strainsifter (top left phylogenetic tree) and average nucleotide identity (ANI; left heatmap). Grayscale binary heatmap shows the presence/absence of antibiotic resistance genes (top right) and virulence factors (bottom left). Side bars depicting phylo group, strain type, AMR mechanism and sample type. For emphasis, the font of isolates from subjects TD-P004 and HT-P041 (mentioned in text) are colored purple and gold, respectively.

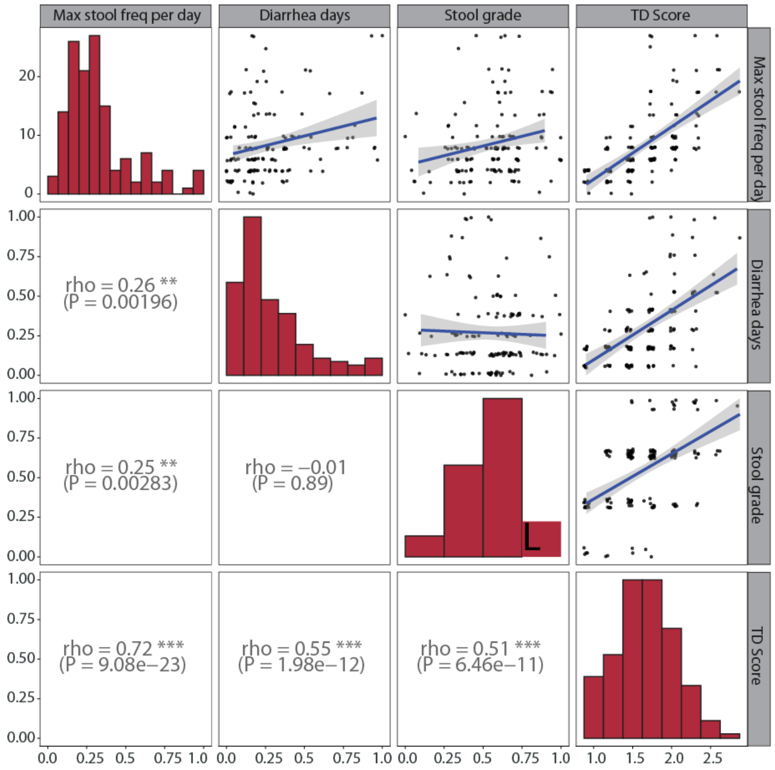

Supplementary Figure 10: Comparison of TD Score with Stool frequency, Stool consistency and Diarrhea duration.

Supplementary Tables

Supplementary Table 1: Description of the participants and the risk factors associated with TD							
**Characteristics**	**Total Obs.**	**All (N = 159)**	**HT (N = 46)**	**TD (N = 113)**	**OR**	**95% CI**	**P-value**
**Demographic factors**							
**Sex**	159						
Female		96 (60%)	30 (65%)	66 (58%)	---	---	
Male		63 (40%)	16 (35%)	47 (42%)	1.34	0.66, 2.77	0.427
**Age**	159	24 (20, 29)	23 (19, 28)	24 (21, 29)	1.02	0.99, 1.06	0.214
**Lodging in cusco**	157						
Other		14 (8.9%)	4 (8.9%)	10 (8.9%)	---	---	
Host Family		52 (33%)	16 (36%)	36 (32%)	0.9	0.22, 3.15	0.874
School Lodging		91 (58%)	25 (56%)	66 (59%)	1.06	0.27, 3.48	0.932
Missing		2	1	1			
**Country**	159						
Others†		25 (16%)	7 (15%)	18 (16%)	---	---	
Canada		10 (6.3%)	4 (8.7%)	6 (5.3%)	0.58	0.12, 2.86	0.492
Germany		21 (13%)	8 (17%)	13 (12%)	0.63	0.18, 2.19	0.468
Netherlands		17 (11%)	4 (8.7%)	13 (12%)	1.26	0.31, 5.67	0.747
Switzerland		19 (12%)	7 (15%)	12 (11%)	0.67	0.18, 2.41	0.534
United Kingdom		14 (8.8%)	3 (6.5%)	11 (9.7%)	1.43	0.32, 7.69	0.653
United States		53 (33%)	13 (28%)	40 (35%)	1.2	0.39, 3.45	0.743
**Previous travel to developing country**	158				1.13	0.54, 2.42	0.751
No		107 (68%)	32 (70%)	75 (67%)			
Yes		51 (32%)	14 (30%)	37 (33%)			
Missing		1	0	1			
**Previous travel in last 1 year**	159				0.8	0.35, 1.72	0.571
No		43 (27%)	11 (24%)	32 (28%)			
Yes		116 (73%)	35 (76%)	81 (72%)			
**Total trip duration (days)**	159	35 (21, 54)	35 (23, 52)	35 (21, 54)	1	0.99, 1.01	0.712
**Preventative measures**							
**Typhoid vaccine**	159				0.73	0.35, 1.49	0.396
No		60 (38%)	15 (33%)	45 (40%)			
Yes		99 (62%)	31 (67%)	68 (60%)			
**HepatitisA vaccine**	144				0.44	0.07, 1.78	0.308
No		12 (8.3%)	2 (4.7%)	10 (9.9%)			
Yes		132 (92%)	41 (95%)	91 (90%)			
Missing		15	3	12			
**Cholera vaccine**	106				2.82	0.96, 10.4	0.08
No		79 (75%)	26 (87%)	53 (70%)			
Yes		27 (25%)	4 (13%)	23 (30%)			
Missing		53	16	37			
**Received any preventative medicine info**	159				1.13	0.45, 2.65	0.782
No		29 (18%)	9 (20%)	20 (18%)			

Yes		130 (82%)	37 (80%)	93 (82%)			
**Health history**							
**Pre-existing bowel disease†**	159				1.55	0.46, 7.08	0.52
No		145 (91%)	43 (93%)	102 (90%)			
Yes		14 (8.8%)	3 (6.5%)	11 (9.7%)			
**GERD or Dyspepsia†**	159				4.17	1.14, 26.9	0.063
No		139 (87%)	44 (96%)	95 (84%)			
Yes		20 (13%)	2 (4.3%)	18 (16%)			
**Lactose intolerant**	159				0.44	0.14, 1.44	0.162
No		146 (92%)	40 (87%)	106 (94%)			
Yes		13 (8.2%)	6 (13%)	7 (6.2%)			
**Other disease†**	159				1.86	0.57, 8.42	0.35
No		143 (90%)	43 (93%)	100 (88%)			
Yes		16 (10%)	3 (6.5%)	13 (12%)			
**Diarrhea in last 2 weeks**	158				1.2	0.57, 2.62	0.632
No		109 (69%)	33 (72%)	76 (68%)			
Yes		49 (31%)	13 (28%)	36 (32%)			
Missing		1	0	1			
**Medication in last 2 weeks for illness or prophylaxis**	158				0.37	0.11, 1.26	0.105
No		147 (92%)	40 (87%)	107 (95%)			
Antibiotic (Ciprofloxacin, Azithromicin, Tetracycline, Trimethoprim)		6 (3.8%)	2 (4.4%)	4 (3.5%)	0.75	0.14, 5.54	0.743
Antidiarrheal (Loperamide hydrochloride)		1 (0.6%)	1 (2.2%)	0 (0%)	0		0.986
Antimalarial (Doxycycline, Malarone)		4 (2.5%)	2 (4.4%)	2 (1.8%)	0.37	0.04, 3.20	0.333
Missing		1	1	0			
**Dietary habits at enrollment**							
**Raw fruits or vegetables**	159				0.89	0.54, 1.52	0.668
Rarely or Never		107 (67%)	30 (65%)	77 (68%)			
Sometimes		37 (23%)	11 (24%)	26 (23%)			
Almost Always or Always		15 (9.4%)	5 (11%)	10 (8.8%)			
**Tap water or unbottled beverages**	159				0.78	0.42, 1.48	0.418
Rarely or Never		135 (85%)	36 (78%)	99 (88%)			
Sometimes		15 (9.4%)	8 (17%)	7 (6.2%)			
Almost Always or Always		9 (5.7%)	2 (4.3%)	7 (6.2%)			
**Under cooked meat**	159				0.93	0.48, 1.90	0.844
Rarely or Never		120 (75%)	34 (74%)	86 (76%)			
Sometimes		35 (22%)	11 (24%)	24 (21%)			
Almost Always or Always		4 (2.5%)	1 (2.2%)	3 (2.7%)			
**Add sauces to food**	159				1.25	0.76, 2.08	0.372
Rarely or Never		41 (26%)	16 (35%)	25 (22%)			
Sometimes		82 (52%)	19 (41%)	63 (56%)			
Almost Always or Always		36 (23%)	11 (24%)	25 (22%)			
**Wash hands before eating**	159				1.04	0.62, 1.69	0.886
Rarely or Never		18 (11%)	6 (13%)	12 (11%)			
Sometimes		45 (28%)	12 (26%)	33 (29%)			
Almost Always or Always		96 (60%)	28 (61%)	68 (60%)			
**Dietary habits at completion**							
**While in peru did you eat raw fruits or vegetables**	159				0.69	0.36, 1.34	0.257

Rarely or Never		128 (81%)	34 (74%)	94 (83%)			
Sometimes		25 (16%)	10 (22%)	15 (13%)			
Almost Always or Always		6 (3.8%)	2 (4.3%)	4 (3.5%)			
**While in peru did you drink local water**	125				0.63	0.28, 1.44	0.256
Rarely or Never		112 (90%)	32 (86%)	80 (91%)			
Sometimes		8 (6.4%)	2 (5.4%)	6 (6.8%)			
Almost Always or Always		5 (4.0%)	3 (8.1%)	2 (2.3%)			
Missing		34	9	25			
**While in peru did you drink unbottled beverages or with ice**	126				1.11	0.61, 2.11	0.734
Rarely or Never		70 (56%)	20 (54%)	50 (56%)			
Sometimes		47 (37%)	16 (43%)	31 (35%)			
Almost Always or Always		9 (7.1%)	1 (2.7%)	8 (9.0%)			
Missing		33	9	24			
**While in peru did you eat raw undercooked meat**	121				0.88	0.29, 2.99	0.826
Rarely or Never		105 (87%)	30 (86%)	75 (87%)			
Sometime		16 (13%)	5 (14%)	11 (13%)			
Missing		38	11	27			
**While in peru did you add sauces to food**	121				0.91	0.54, 1.54	0.724
Rarely or Never		38 (31%)	10 (29%)	28 (33%)			
Sometimes		53 (44%)	16 (46%)	37 (43%)			
Almost Always or Always		30 (25%)	9 (26%)	21 (24%)			
Missing		38	11	27			
**While in peru did you wash hands before eating**	121				0.81	0.40, 1.51	0.515
Rarely or Never		9 (7.4%)	3 (8.6%)	6 (7.0%)			
Sometimes		34 (28%)	7 (20%)	27 (31%)			
Almost Always or Always		78 (64%)	25 (71%)	53 (62%)			
Missing		38	11	27			

Statistics presented: Median (IQR) for 'Age' and 'Total duration in days' ; n (%) for all other categorial variables

p-value = Estimated by univariate logistic regression

q-value = False discovery rate correction for multiple testing

OR = Odds Ratio, CI = Confidence Interval

HT = Healthy Travelers

TD = Travelers Diarrhea

GERD† = Gastroesophageal reflux disease

Pre-existing bowel disease† = ‘Diarrhea-predominant IBS’, ‘Constipation-predominant IBS’, ‘Functional diarrhea’, ‘Functional Constipation’

Other countries† = 'Australia', 'Austria', 'Brazil', 'Belgium', 'France', 'Hong Kong', 'Israel', 'Italy', 'Norway', 'Sweden'

Other diseases† = ‘Asthma’, ‘Insomnia’, ‘Narclepsy’, ‘Migranes’, ‘Hypothyrodism’, ‘Osteoarthritis’

Supplementary Table 2: Description of samples and dietary habits during weekly follow-up

Characteristic	Total Obs.	Overall (N = 718)	ND (N = 574)	D (N = 144)	OR	95% CI	p-value	q-value
**Stool_grade**	718				**21.6**	13.1, 35.4	<0.001	**<0.001**
Grade-1		424 (59%)	415 (72%)	9 (6.2%)				
Grade-2		193 (27%)	151 (26%)	42 (29%)				
Grade-3		84 (12%)	8 (1.4%)	76 (53%)				
Grade-4		17 (2.4%)	0 (0%)	17 (12%)				
**Antibiotics in last week**	85	85 (24%)	61 (21%)	24 (41%)	**2.55**	1.41, 4.61	0.002	**0.007**
Missing		371	286	85				
**Location of majority of meals in past week**								
**Street vendors**	439				0.44	0.19, 1.03	0.057	0.1
Rarely or Never		362 (82%)	303 (81%)	59 (92%)				
Sometimes		70 (16%)	66 (18%)	4 (6.2%)				
Amost Always or Always		7 (1.6%)	6 (1.6%)	1 (1.6%)				
Missing		279	199	80				
**Self-prepared food**	445				0.63	0.41, 0.95	0.027	0.064
Rarely or Never		227 (51%)	185 (49%)	42 (66%)				
Sometimes		139 (31%)	125 (33%)	14 (22%)				
Amost Always or Always		79 (18%)	71 (19%)	8 (12%)				
Missing		273	193	80				
**Local cheap restaurants**	448				0.99	0.68, 1.45	0.978	0.978
Rarely or Never		115 (26%)	99 (26%)	16 (25%)				
Sometimes		224 (50%)	191 (50%)	33 (52%)				
Amost Always or Always		109 (24%)	94 (24%)	15 (23%)				
Missing		270	190	80				
**Expensive restaurants**	446				1.23	0.79, 1.92	0.358	0.502
Rarely or Never		281 (63%)	241 (63%)	40 (61%)				
Sometimes		147 (33%)	126 (33%)	21 (32%)				
Amost Always or Always		18 (4.0%)	13 (3.4%)	5 (7.6%)				
Missing		272	194	78				
**Amauta kitchen**	448				0.91	0.68, 1.20	0.495	0.577
Rarely or Never		168 (38%)	141 (37%)	27 (42%)				
Sometimes		51 (11%)	45 (12%)	6 (9.4%)				
Amost Always or Always		229 (51%)	198 (52%)	31 (48%)				
Missing		270	190	80				

Statistics presented: n (%) for all other categorical variables

ND = Non-diarrhea samples

D = Diarrhea samples

OR = Odds Ratio, CI = Confidence Interval
CI = Confidence interval

p-value = Estimated by univariate logistic regression with 'subject' as random effect

q-value = False discovery rate correction for multiple testing

Supplementary Table 3: Features with systematic effects on the microbial community using PERMANOVA with repeated measures

Category	Features	Taxonomic Profile using Metaphlan2				Resistome Profile using ShortBRED			
		R2	*Pr(>F)*	*R2 (%)*	*P.adj*	*R2*	*Pr(>F)*	*R2 (%)*	*P.adj*
Demographics	**Duration of stay (Weeks)**	0.009	0.007	0.897	**0.020**	0.011	0.012	1.143	**0.035**
Demographics	Lodging (in Cusco)	0.009	0.268	0.916	0.412	0.007	0.499	0.735	0.641
Demographics	Previous travel	0.004	0.440	0.405	0.586	0.010	0.026	0.961	0.064
Demographics	Traveler type	0.006	0.118	0.566	0.214	0.006	0.143	0.624	0.286
Demographics	**Country of origin**	0.039	0.000	3.884	**0.001**	0.034	0.000	3.408	**0.001**
Demographics	Age	0.009	0.011	0.872	**0.028**	0.003	0.648	0.292	0.720
Demographics	Sex	0.007	0.042	0.686	0.094	0.005	0.275	0.470	0.458
Sample Characteristics	**Stool grade**	0.016	0.000	1.555	**0.001**	0.013	0.001	1.299	**0.002**
Sample Characteristics	**Sample type (Non-diarrhea/Diarrhea)**	0.012	0.000	1.168	**0.001**	0.009	0.000	0.856	**0.001**
Sample Characteristics	**Sample Collection (Weeks)**	0.005	0.532	0.481	0.665	0.014	0.000	1.350	**0.001**
Sample Characteristics	**Pathogen presence**	0.004	0.002	0.403	**0.007**	0.004	0.049	0.374	0.108
Vaccines	Typhoid	0.003	0.739	0.309	0.739	0.002	0.972	0.169	0.972
Vaccines	Cholera	0.006	0.396	0.647	0.566	0.006	0.497	0.570	0.641
Vaccines	Hepatitis A	0.004	0.624	0.362	0.672	0.003	0.572	0.339	0.673
Disease	GERD	0.006	0.096	0.592	0.193	0.004	0.447	0.363	0.641
Disease	Dyspepsia	0.003	0.638	0.325	0.672	0.002	0.824	0.222	0.868
Disease	Lactose intolerance	0.005	0.209	0.507	0.348	0.003	0.513	0.329	0.641
Disease	Pre-existing bowel disease	0.003	0.628	0.331	0.672	0.005	0.187	0.547	0.340
	**Subject**	0.517	0.000	51.665	**0.001**	0.505	0.000	50.476	**0.001**
	**Overall**	0.536	0.000	53.626	**0.001**	0.533	0.000	53.288	**0.001**

Supplementary Table 4a: Change in alpha-diversity over time in HT and TD subjects after adjusting for age, gender and region

	Richness				Shannon diversity index			
term	*estimate*	*std.error*	*statistic*	*p.value*	*estimate*	*std.error*	*statistic*	*p.value*
(Intercept)	4.009	0.058	69.021	0.000	2.732	0.109	25.062	0.000
Abx_dur_stay[Yes]	-0.040	0.033	-1.214	0.225	-0.023	0.060	-0.393	0.695
Age	0.016	0.014	1.121	0.262	0.021	0.025	0.832	0.407
Gender[Male]	0.005	0.027	0.197	0.844	0.007	0.050	0.136	0.892
Time_in_Peru_days	-0.001	0.020	-0.042	0.967	0.014	0.047	0.289	0.773
Region[Europe]	0.050	0.053	0.939	0.348	-0.044	0.099	-0.444	0.657
Region[North America]	0.034	0.054	0.627	0.531	-0.089	0.099	-0.901	0.369
Sample_type[Diarrhea TD]	-0.047	0.042	-1.117	0.264	-0.118	0.089	-1.326	0.185
**Sample_type[Diarrhea TD:Time_in_Peru_days]**	0.078	0.038	2.074	**0.038**	0.138	0.090	1.544	0.123
**Sample_type[Non-diarrhea PostTD]**	-0.128	0.046	-2.801	**0.005**	-0.142	0.097	-1.474	0.141
**Sample_type[Non-diarrhea PostTD:Time_in_Peru_days]**	0.083	0.033	2.523	**0.012**	0.088	0.077	1.142	0.254
Sample_type[Non-diarrhea PreTD]	0.004	0.038	0.112	0.911	-0.060	0.079	-0.757	0.450
Sample_type[Non-diarrhea PreTD:Time_in_Peru_days]	0.014	0.034	0.399	0.690	0.020	0.080	0.255	0.799

*formula = Response [Richness|Shannon] ~ Sample_type * Length_of_stay_in_days + Age + Gender + Region + Abx_dur_stay + (1 | Subject_id)*
Linear mixed effect mode (LMM) was used to fit **Shannon index** using `lmer` function of lmerTest package in R.
Generalized linear mixed model (GLMM) was used to fit **Richness** using glmer (family = poisson) function of lme4 package in R

Supplementary Table 4b: Alpha-diversity comparison of diarrhea samples with matched non-diarrhea samples (Pre TD and Post TD)

	Richness				Shannon diversity index			
term	*estimate*	*std.error*	*statistic*	*adj.p.value*	*estimate*	*std.error*	*statistic*	*adj.p.value*
**Non-diarrhea PostTD - Diarrhea TD**	-0.146	0.026	-5.552	**0.000**	-0.205	0.081	-2.531	**0.038**
**Non-diarrhea PostTD - Non-diarrhea PreTD**	-0.156	0.032	-4.820	**0.000**	-0.195	0.095	-2.048	0.064
**Non-diarrhea PreTD - Diarrhea TD**	0.010	0.026	0.373	0.709	-0.011	0.085	-0.123	0.902

formula = Response [Richness|Shannon] ~ Sample_type + Age + Gender + Region + Abx_dur_stay + Length_of_stay_in_days + (1 | Subject_id)

Results are averaged over the levels of: Sex, Region, Abx_dur_stay

P value adjustment: **FDR** method for 3 tests

Supplementary Table 5a: Features associated with intra-subject microbial stabilty over time

	1 - Bray-curtis index				1 - Jaccard index			
term	*estimate*	*std.error*	*statistic*	*p.value*	*estimate*	*std.error*	*statistic*	*p.value*
(Intercept)	0.399	0.087	4.595	0.000	0.301	0.092	3.292	0.002
Abx_dur_stay[Yes]	0.032	0.029	1.124	0.267	0.030	0.030	0.991	0.327
Age	0.000	0.011	0.030	0.976	0.002	0.012	0.179	0.859
**Baseline_Shannon**	0.359	0.064	5.647	**0.000**	0.333	0.067	4.952	**0.000**
Sex[Male]	0.019	0.025	0.761	0.450	0.013	0.026	0.498	0.620
Time_in_Peru_days	-0.014	0.019	-0.742	0.459	-0.017	0.020	-0.883	0.378
Region[Europe]	-0.047	0.060	-0.798	0.427	-0.069	0.063	-1.102	0.274
Region[North America]	-0.039	0.059	-0.659	0.511	-0.060	0.062	-0.973	0.333
**Traveler_type[TD]**	-0.083	0.023	-3.581	**0.001**	-0.086	0.024	-3.514	**0.001**

formula = Response [Bray-Curtis|Jaccard] ~ Traveler_type + Time_in_Peru_days + Baseline_Shannon + Age + Sex + Region + Abx_dur_stay + (1 | Subject_id)

Linear mixed effect mode (LMM) was used to fit Bray-Curtis and Jaccard index using `lmer` function of lmerTest package in R.

Supplementary Table 5b: Features associated with taxonomic divergence (comparing beta-diversity of each traveler's samples to first week baseline sample)

	1 - Bray-curtis index				1 - Jaccard index			
term	*estimate*	*std.error*	*statistic*	*p.value*	*estimate*	*std.error*	*statistic*	*p.value*
(Intercept)	0.348	0.125	2.777	0.008	0.272	0.124	2.188	0.034
Abx_dur_stay[Yes]	0.017	0.040	0.428	0.671	0.011	0.040	0.286	0.776
Age	0.009	0.016	0.526	0.602	0.011	0.016	0.679	0.501
**Baseline_Shannon**	0.454	0.107	4.221	**0.000**	0.392	0.107	3.672	**0.001**
Sex[Male]	0.002	0.034	0.057	0.955	0.002	0.034	0.056	0.956
**Region[Europe]**	-0.143	0.074	-1.944	0.058	-0.158	0.073	-2.161	**0.036**
Region[North America]	-0.124	0.071	-1.745	0.088	-0.137	0.070	-1.942	0.058
**Time_diff_days**	-0.076	0.016	-4.895	**0.000**	-0.079	0.015	-5.099	**0.000**
Traveler_type[TD]	-0.021	0.034	-0.621	0.538	-0.024	0.034	-0.713	0.480

formula = Response [Bray-Curtis|Jaccard] ~ Traveler_type + Time_diff_days + Baseline_Shannon + Age + Sex + Region + Abx_dur_stay + (1 | Subject_id)

Linear mixed effect mode (LMM) was used to fit Bray-Curtis and Jaccard index using `lmer` function of lmerTest package in R.

Supplementary Table 6: Summary of microbiome shift events

		Microbiome shifts		
Traveler type	*Sample type*	*Total Possible*	*Observed*	*Rate (%)*
HT	A-A	128	28	21.90%
TD	A-A	147	37	25.20%
TD	A-D	120	68	56.70%
TD	D-D	13	8	61.50%

Supplementary Table 7: Summary of the differential network analysis.										
S_ID	***n (cntrl)***	***n (diar)***	***core (diar)***	***Union***	***Intersect***	***Exclusive***	***Jaccard-score***	***NESH-score***	***DelBet***	***COM***
Ruminococcus_torques	1	6	3	6	1	5	0.167	2.381	0.542	1
Eubacterium_rectale	3	5	3	8	0	5	0	2.339	0.125	3
Bacteroides_uniformis	2	6	2	7	1	5	0.143	2.286	0.331	4
Methanobrevibacter_smithii	1	2	1	3	0	2	0	1.952	0.206	1
Roseburia_inulinivorans	1	2	2	3	0	2	0	1.952	0.125	1
Coprococcus_comes	3	3	3	6	0	3	0	1.929	-0.055	2
Bifidobacterium_longum	4	6	3	8	2	4	0.25	1.821	0.751	1
Bifidobacterium_adolescentis	3	4	2	6	1	3	0.167	1.762	0.553	1
Bifidobacterium_pseudocatenulatum	1	1	1	2	0	1	0	1.643	0	1
Clostridium_bartlettii	1	1	1	2	0	1	0	1.643	0	1
Bacteroides_caccae	1	3	2	3	1	2	0.333	1.619	0.227	4
Subdoligranulum_unclassified	3	3	2	5	1	2	0.2	1.486	-0.013	5
Ruminococcus_bromii	2	1	1	3	0	1	0	1.476	-0.34	1
Ruminococcus_lactaris	2	1	1	3	0	1	0	1.476	0	3
Collinsella_aerofaciens	4	6	3	7	3	3	0.429	1.429	0.686	2
Dorea_formicigenerans	5	6	3	8	3	3	0.375	1.429	0.414	3
Faecalibacterium_prausnitzii	7	3	2	9	1	2	0.111	1.397	-0.866	5
Eubacterium_ramulus	3	1	1	4	0	1	0	1.393	-0.129	3
Alistipes_putredinis	1	2	2	2	1	1	0.5	1.143	0	4
Bacteroides_dorei	3	2	2	4	1	1	0.25	1.143	-0.107	2
Escherichia_coli	1	2	2	2	1	1	0.5	1.143	0.023	2
Escherichia_unclassified	1	2	2	2	1	1	0.5	1.143	0.134	2
Dorea_longicatena	6	7	3	8	5	2	0.625	0.911	0.264	3
Eubacterium_hallii	6	4	3	7	3	1	0.429	0.857	-0.461	3
Ruminococcus_sp_5_1_39BFAA	5	5	3	6	4	1	0.667	0.643	0.093	3
Ruminococcus_gnavus	5	2	2	5	2	0	0.4	0.6	-1	5
Bacteroides_vulgatus	5	3	2	5	3	0	0.6	0.4	-0.377	4
Lachnospiraceae_bacterium_1_1_57FAA	1	1	1	1	1	0	1	0	0	1
Ruminococcus_obeum	4	4	3	4	4	0	1	0	0.028	3
Streptococcus_parasanguinis	1	1	1	1	1	0	1	0	0	6
Streptococcus_salivarius	1	1	1	1	1	0	1	0	0	6

n(control/case) referes to degree of the node in control/case, Exclusive refers to exclusive in 'case', DelBet refers to the delta betweenness score from control to case and COM refers to the community affiliation of the node in 'case'.

Supplementary Table 8: Functionally validated antibiotic resistance gene sequences from previously published cohort that were used to build ShortBRED markers

Num	Study_Title	Pubmed ID	# of Library	# of Abx	# of Slxns	Uniq_AR_ProteinSeqs
1	The shared antibiotic resistome of soil bacteria and human pathogens	22936781	1	16	16	54
2	Pediatric fecal microbiota harbor diverse and novel antibiotic resistance genes	24236055	22	13	169	2536
3	Bacterial phylogeny structures soil resistomes across habitats	24847883	18	15	219	2527
4	Gut resistome development in healthy twin pairs in the first year of life	26113976	26	16	195	1383
5	The microbiome of uncontacted Amerindians	26229982	16	10	52	106
6	Developmental dynamics of the preterm infant gut microbiota and antibiotic resistome	27572443	21	11	182	1129
7	Interconnected microbiomes and resistomes in low-income human habitats	27172044	79	16	546	1915
8	Characterization of Wild and Captive Baboon Gut Microbiota and Their Antibiotic Resistomes	29963641	8	9	43	382
9	Persistent metagenomic signatures of early-life hospitalization and antibiotic treatment in the infant gut microbiota and resistome	31501537	22	15	262	1404
10	**Impact of international travel and diarrhea on gut microbiome and resistome dynamics**	**Current study**	21	17	388	2065
11	Manure Microbial Communities and Resistance Profiles Reconfigure after Transition to Manure Pits and Differ from Those in Fertilized Field Soil	33975936	9	17	153	2235
12	Destination shapes antibiotic resistance gene acquisitions, abundance increases, and diversity changes in Dutch travelers	34092249	21	15	234	1443
13	The microbiome and resistome of chimpanzees, gorillas, and humans across host lifestyle and geography	32203121	17	14	142	323
		**TOTAL**	**281**	**14**	**2367**	**17502**

Supplementary Table 9a: Change in alpha-diversity and cumulative abundance of ARGs over time

	Richness				Shannon diversity index				Cumulative abundance log10(RPKM)			
term	*estimate*	*std.error*	*statistic*	*p.value*	*estimate*	*std. error*	*statistic*	*p.value*	*estimate*	*std. error*	*statistic*	*p.value*
(Intercept)	3.742	0.088	42.462	0.000	2.999	0.116	25.881	0.000	3.025	0.087	34.575	0.000
Abx_dur_stay[Yes]	-0.091	0.051	-1.790	0.073	-0.023	0.061	-0.371	0.711	-0.041	0.050	-0.810	0.420
Age	0.038	0.021	1.761	0.078	0.059	0.026	2.265	**0.025**	0.017	0.021	0.780	0.437
Sex[Male]	-0.025	0.043	-0.597	0.550	-0.079	0.051	-1.527	0.129	0.004	0.042	0.088	0.930
Time_in_Peru_days	0.034	0.023	1.459	0.145	0.015	0.055	0.280	0.780	0.023	0.028	0.843	0.399
Region[Europe]	0.064	0.081	0.788	0.431	-0.119	0.104	-1.144	0.254	0.086	0.080	1.071	0.286
Region[North America]	0.088	0.082	1.068	0.286	-0.064	0.105	-0.611	0.542	0.090	0.081	1.113	0.268
Sample_type[Diarrhea TD]	0.009	0.058	0.162	0.871	-0.014	0.100	-0.136	0.892	0.105	0.062	1.696	0.091
**Sample_type[Diarrhea TD]:Time_in_Peru_days**	0.110	0.043	2.581	**0.010**	0.113	0.106	1.073	0.284	0.040	0.053	0.757	0.450
Sample_type[Non-diarrhea PostTD]	-0.063	0.062	-1.013	0.311	-0.159	0.109	-1.456	0.146	0.102	0.066	1.537	0.125
Sample_type[Non-diarrhea PostTD:Time_in_Peru_days]	0.007	0.038	0.190	0.849	0.030	0.090	0.336	0.737	-0.017	0.045	-0.378	0.706
Sample_type[Non-diarrhea PreTD]	-0.016	0.054	-0.297	0.766	-0.081	0.088	-0.923	0.357	0.056	0.057	0.986	0.325
Sample_type[Non-diarrhea PreTD:Time_in_Peru_days]	-0.073	0.040	-1.822	0.068	-0.043	0.094	-0.456	0.649	-0.058	0.047	-1.220	0.223

*formula = Response [Richness|Shannon | log10(RPKM)] ~ Sample_type * Length_of_stay_in_days + Age + Sex + Region + Abx_dur_stay + (1 | Subject_id)*

Linear mixed effect mode (LMM) was used to fit **Shannon index and RPKM** using `lmer` function of lmerTest package in R.
Generalized linear mixed model (GLMM) was used to fit **Richness** using glmer (family = poisson) function of lme4 package in R

Supplementary Table 9b: Prior week antibiotics use signficantly associated with increased richness and abundance of ARGs

	Richness				Shannon diversity index				Cumulative abundance log10(RPKM)			
term	*estimate*	*std.error*	*statistic*	*p.value*	*estimate*	*std.error*	*statistic*	*p.value*	*estimate*	*std.error*	*statistic*	*p.value*
(Intercept)	3.589	0.109	33.050	0.000	2.891	0.137	21.033	0.000	3.037	0.095	31.969	0.000
Age	-0.002	0.028	-0.056	0.955	0.027	0.031	0.855	0.396	0.000	0.024	-0.010	0.992
**Antibiotics_in_last_week[Yes]**	0.140	0.028	5.004	**0.000**	0.047	0.067	0.706	0.481	0.101	0.034	2.993	**0.003**
SexMale	-0.031	0.060	-0.517	0.605	-0.110	0.070	-1.560	0.122	0.020	0.052	0.390	0.697
RegionEurope	0.187	0.109	1.722	0.085	-0.011	0.137	-0.077	0.938	0.109	0.095	1.151	0.252
RegionNorth America	0.209	0.110	1.898	0.058	0.025	0.138	0.181	0.857	0.102	0.096	1.061	0.291

formula = Response [Richness|Shannon | log10(RPKM)] ~ Antibiotics_in_last_week + Age + Sex + Region + (1 | Subject_id)
Linear mixed model fit by REML. t-tests use Satterthwaite's method ['lmerModLmerTest']
Linear mixed effect mode (LMM) was used to fit **Shannon index and RPKM** using `lmer` function of lmerTest package in R.
Generalized linear mixed model (GLMM) was used to fit **Richness** using glmer (family = poisson) function of lme4 package in R

Supplementary Table 9c: Increase in alpha-diversity and cumulative abundance of antibiotic resistance genes during diarrheal event

	Richness				Shannon diversity index				Cumulative abundance log10(RPKM)			
term	*estimate*	*std. error*	*statistic*	*adj. p.value*	*estimate*	*std. error*	*statistic*	*adj. p.value*	*estimate*	*std. error*	*statistic*	*adj. p.value*
**Diarrhea TD - Non-diarrhea PreTD**	0.221	0.031	7.177	**0.000**	0.269	0.094	2.879	**0.014**	0.143	0.044	3.227	**0.005**
**Non-diarrhea PostTD - Non-diarrhea PreTD**	0.041	0.039	1.034	0.301	0.038	0.103	0.369	0.712	0.127	0.053	2.403	**0.026**
**Non-diarrhea PostTD - Diarrhea TD**	-0.180	0.029	-6.150	**0.000**	-0.231	0.089	-2.598	**0.016**	-0.015	0.042	-0.364	0.716

formula = Response [Richness|Shannon | log10(RPKM)] ~ Sample_type + Age + Sex+ Region + Abx_dur_stay + Length_of_stay_in_days + (1 | Subject_id)

Results are averaged over the levels of: Sex, Region, Abx_dur_stay

P value adjustment: **FDR** method for 3 tests

Supplementary Table 10a: Intra-subject resistome stabilty over time

	1 - Bray-curtis index				1 - Jaccard index			
term	*estimate*	*std.error*	*statistic*	*p.value*	*estimate*	*std.error*	*statistic*	*p.value*
(Intercept)	0.617	0.105	5.852	0.000	0.480	0.101	4.756	0.000
Abx_dur_stay[Yes]	0.049	0.033	1.475	0.145	0.043	0.032	1.374	0.174
Age	0.014	0.012	1.105	0.273	0.014	0.012	1.197	0.235
Baseline_Shannon	0.080	0.082	0.970	0.335	0.058	0.078	0.743	0.460
Sex[Male]	-0.014	0.027	-0.519	0.606	-0.016	0.026	-0.611	0.543
**Time_in_Peru_days**	0.028	0.015	1.834	0.068	0.030	0.015	2.027	**0.044**
Region[Europe]	-0.095	0.065	-1.462	0.147	-0.099	0.062	-1.591	0.115
Region[North America]	-0.108	0.064	-1.702	0.092	-0.109	0.061	-1.788	0.077
**Traveler_type[TD]**	-0.052	0.026	-2.049	**0.044**	-0.051	0.024	-2.064	**0.043**

formula = [Bray-curtis | Jaccard] ~ Traveler_type + Length_of_stay_in_days + Baseline_Shannon + Age + Sex + Region + Abx_dur_stay + (1 | Subject_id)
Linear mixed model fit by REML. t-tests use Satterthwaite's method ['lmerModLmerTest']

Supplementary Table 10b: Comparing beta-diversity of each traveler's samples to first week baseline sample

	1 - Bray-curtis index				1 - Jaccard index			
term	*estimate*	*std.error*	*statistic*	*p.value*	*estimate*	*std.error*	*statistic*	*p.value*
(Intercept)	0.703	0.103	6.831	0.000	0.561	0.095	5.932	0.000
Abx_dur_stay[Yes]	0.010	0.035	0.296	0.769	0.008	0.032	0.236	0.815
Age	0.022	0.014	1.578	0.122	0.024	0.013	1.836	0.073
Baseline_Shannon	0.009	0.085	0.110	0.913	-0.003	0.078	-0.033	0.974
Sex[Male]	0.023	0.030	0.766	0.448	0.019	0.027	0.676	0.502
Region[Europe]	-0.107	0.064	-1.672	0.100	-0.118	0.059	-2.006	**0.050**
Region[North America]	-0.120	0.062	-1.950	0.056	-0.127	0.057	-2.242	**0.029**
**Time_diff_days**	-0.036	0.014	-2.593	**0.010**	-0.037	0.013	-2.837	**0.005**
Traveler_type[TD]	-0.037	0.030	-1.241	0.221	-0.033	0.027	-1.195	0.238

formula = [Bray-curtis | Jaccard] ~ Traveler_type + Time_diff_days + Baseline_Shannon + Age + Sex + Region + Abx_dur_stay + (1 | Subject_id)
Linear mixed model fit by REML. t-tests use Satterthwaite's method ['lmerModLmerTest']

Supplementary Table 11: Description of enteric pathogens detected using multiplex PCR

Characteristic	Overall N = 718	Non-diarrhea N = 574	Diarrhea N = 144	OR	95% CI	p-value	q-value
**Diarrheagenic *E.coli (DEC)***							
EAEC	47 (6.9%)	40 (7.4%)	7 (5.2%)	0.69	0.30, 1.57	0.372	0.496
EPEC	48 (7.1%)	38 (7.0%)	10 (7.4%)	1.06	0.51, 2.19	0.872	0.872
ETEC	39 (5.8%)	22 (4.1%)	17 (13%)	3.42	1.70, 6.88	**<0.001**	**0.002**
DAEC	33 (4.9%)	26 (4.8%)	7 (5.2%)	1.09	0.46, 2.56	0.851	0.872
STEC	18 (2.7%)	12 (2.2%)	6 (4.4%)	2.05	0.76, 5.58	0.158	0.237
EIEC	4 (0.6%)	3 (0.6%)	1 (0.7%)	1.34	0.14, 13.0	0.8	0.872
**Other bacterial pathogens**							
Campylobacter	18 (2.6%)	7 (1.3%)	11 (7.7%)	6.57	2.50, 17.3	**<0.001**	**0.002**
Shigella	3 (0.4%)	1 (0.2%)	2 (1.4%)	7.91	0.71, 87.9	0.092	0.158
**Viral pathogens**							
Norovirus	29 (4.2%)	18 (3.2%)	11 (7.7%)	2.51	1.16, 5.43	**0.02**	**0.04**
**Infection type**							
No pathogen detected	479 (69%)	399 (72%)	80 (56%)	0.5	0.34, 0.74	**<0.001**	**0.002**
Mixed pathogens detected	20 (2.8%)	11 (1.9%)	9 (6.2%)	3.41	1.39, 8.40	**0.008**	**0.018**
Any pathogen detected	217 (31%)	155 (28%)	62 (44%)	2	1.35, 2.96	**<0.001**	**0.002**

Statistics presented: n (%) for all other categorical variables

p-value = Estimated by univariate logistic regression with 'subject' as random effect

q-value = False discovery rate correction for multiple testing

OR = Odds Ratio, CI = Confidence Interval

Supplementary Table 12: AST profile of DEC isolates

			Diarrheagenic E.coli strains			
**Abx_class**	**Abx_name**	**Abx_code**	**Resistant**	**Intermediate**	**Susceptible**	**Missing**
Aminoglycoside	*Amikacin*	AMK	0 (0%)	0 (0%)	169 (100%)	0 (0%)
Beta_lactam	*Ceftazidime*	CAZ	0 (0%)	0 (0%)	150 (88.8%)	19 (11.2%)
Beta_lactam	*Cefotaxime - Clavulanate*	CTX_CLA	0 (0%)	2 (1.2%)	148 (87.6%)	19 (11.2%)
Beta_lactam	*Imipenem*	IPM	0 (0%)	0 (0%)	169 (100%)	0 (0%)
Nitrofurans	*Nitrofurantoin*	NIT	0 (0%)	0 (0%)	150 (88.8%)	19 (11.2%)
Aminoglycoside	*Gentamycin*	GEN	6 (3.6%)	0 (0%)	163 (96.4%)	0 (0%)
Amphenicol	*Chloramphenical*	CHL	20 (11.8%)	1 (0.6%)	147 (87%)	1 (0.6%)
Beta_lactam	*Ticarcillin - Clavulanic Acid*	TIM	64 (37.9%)	8 (4.7%)	78 (46.2%)	19 (11.2%)
Beta_lactam	*Amoxicillin - Clavulanate*	AMX_CLA	30 (17.8%)	39 (23.1%)	99 (58.6%)	1 (0.6%)
Beta_lactam	*Cefalotin*	CEF	26 (15.4%)	55 (32.5%)	88 (52.1%)	0 (0%)
Beta_lactam	*Ceftriaxone*	CRO	3 (1.8%)	2 (1.2%)	164 (97%)	0 (0%)
Beta_lactam	*Cefepime*	FEP	1 (0.6%)	1 (0.6%)	167 (98.8%)	0 (0%)
Macrolide	*Azithromycin*	AZM	40 (23.7%)	33 (19.5%)	95 (56.2%)	1 (0.6%)
Quinolone	*Nalidixic Acid*	NAL	49 (29%)	14 (8.3%)	104 (61.5%)	2 (1.2%)
Quinolone	*Ciprofloxacin*	CIP	13 (7.7%)	18 (10.7%)	138 (81.7%)	0 (0%)
Beta_lactam	*Ampicillin*	AMP	111 (65.7%)	9 (5.3%)	48 (28.4%)	1 (0.6%)
Beta_lactam	*Ticarcillin*	TIC	103 (60.9%)	2 (1.2%)	59 (34.9%)	5 (3%)
Folate_synthesis_inhibitor	*Trimethoprim-sulphamethoxazole*	SXT	90 (53.3%)	2 (1.2%)	56 (33.1%)	21 (12.4%)
Macrolide	*Erythromycin*	ERY	162 (95.9%)	7 (4.1%)	0 (0%)	0 (0%)
Tetracycline	*Tetracycline*	TET	98 (58%)	1 (0.6%)	70 (41.4%)	0 (0%)

Supplementary Table 13: Prevalence of ARGs in DEC isolates				
AMR mechanism	*AMR family*	*isCore gene*	*Total cnt*	*Prev*
Antibiotic inactivation	ampC beta lactamase	Yes	189	100.00%
Antibiotic efflux	MacAB tolC ABC Transporter	Yes	189	100.00%
Antibiotic efflux	msbA ABC Transporter	Yes	189	100.00%
Antibiotic efflux	mdtNOP MFS efflux pump	Yes	189	100.00%
Antibiotic efflux	mdtH MFS efflux pump	Yes	189	100.00%
Antibiotic efflux	mdtABC tolC RND efflux pump	Yes	189	100.00%
Antibiotic efflux	mdfA MFS efflux pump	Yes	189	100.00%
Antibiotic efflux	EmrKY tolC MFS efflux pump	Yes	189	100.00%
Antibiotic efflux	EmrAB tolC MFS efflux pump	Yes	189	100.00%
Antibiotic efflux	acrD RND efflux pump	Yes	189	100.00%
Antibiotic efflux	AcrAB tolC RND efflux pump	Yes	189	100.00%
Antibiotic efflux	Yoji ABC Transporter	Yes	189	100.00%
Antibiotic target alteration	Undecaprenyl pyrophosphate related proteins	Yes	189	100.00%
Antibiotic target alteration	Pmr phosphoethanolamine transferase	Yes	189	100.00%
Antibiotic inactivation	BlaEC beta lactamase	Yes	188	99.47%
Antibiotic efflux	Unknown ABC Transporter	Yes	188	99.47%
Antibiotic efflux	mdtEF tolC RND efflux pump	Yes	188	99.47%
Antibiotic efflux	mdtG MFS efflux pump	Yes	187	98.94%
Antibiotic efflux	AcrEF tolC RND efflux pump	Yes	184	97.35%
Antibiotic inactivation	MphB macrolide phosphotransferase	Yes	181	95.77%
Antibiotic efflux	mdtM MFS efflux pump	Yes	180	95.24%
Antibiotic efflux	emrE SMR efflux pump	No	115	60.85%
Antibiotic target replacement	sul sulfonamide resistant dihydropteroate synthase	No	88	46.56%
Antibiotic inactivation	TEM beta lactamase	No	82	43.39%
Antibiotic target replacement	dhfr	No	76	40.21%
Antibiotic inactivation	APH6	No	66	34.92%
Antibiotic inactivation	APH3	No	64	33.86%
Antibiotic target alteration	fluoroquinolone resistant gyrA	No	48	25.40%
Antibiotic efflux	tetA MFS efflux pump	No	46	24.34%
Antibiotic inactivation	ANT3	No	36	19.05%
Antibiotic efflux	qac MFS efflux pump	No	36	19.05%
Antibiotic efflux	tetAB ABC Transporter	No	31	16.40%
Antibiotic target protection	qnrB Quinolone resistance protein	No	25	13.23%
Antibiotic inactivation	catA chloramphenicol acetyltransferase	No	21	11.11%
Antibiotic inactivation	MphA macrolide phosphotransferase	No	21	11.11%
Antibiotic efflux	Unknown RND efflux pump	No	18	9.52%
Antibiotic efflux	mexEF OprN RND efflux pump	No	17	8.99%
Antibiotic target alteration	fluoroquinolone resistant parC	No	13	6.88%
Antibiotic target protection	Unknown ABC F proteins	No	11	5.82%
Antibiotic inactivation	sat2 streptothricin acetyltransferase	No	10	5.29%
Antibiotic target alteration	fluoroquinolone resistant parE	No	9	4.76%
Antibiotic target alteration	Erm 23S rRNA Methyltransferase	No	8	4.23%
Antibiotic inactivation	Unknown Class B beta lactamase	No	6	3.17%
Antibiotic inactivation	CTX M beta lactamase	No	6	3.17%

Antibiotic inactivation	OXA beta lactamase	No	4	2.12%
Antibiotic inactivation	AAC3	No	3	1.59%
Antibiotic inactivation	FosA Fosfomycin thiol transferase	No	3	1.59%
Antibiotic target protection	qnrS Quinolone resistance protein	No	3	1.59%
Antibiotic efflux	qepA MFS efflux pump	No	2	1.06%
Antibiotic inactivation	AAC6	No	1	0.53%
Antibiotic efflux	flo MFS efflux pump	No	1	0.53%
Antibiotic efflux	cmlA MFS efflux pump	No	1	0.53%
Antibiotic inactivation	Unknown Class A beta lactamase	No	1	0.53%
Antibiotic inactivation	CMY beta lactamase	No	1	0.53%
Antibiotic inactivation	CARB beta lactamase	No	1	0.53%
Antibiotic inactivation	Bleomycin Resistant Protein	No	1	0.53%
Antibiotic efflux	Unknown MFS efflux pump	No	1	0.53%
Antibiotic efflux	oqxAB RND efflux pump	No	1	0.53%
Antibiotic target alteration	MCR phosphoethanolamine transferase	No	1	0.53%
Antibiotic target protection	qnrA Quinolone resistance protein	No	1	0.53%

Supplementary Table 14: Modified scoring scheme for diarrhea sample evaluation				
		**1 point**	**2 point**	**3 point**
A	***High Level of care, Fever, Blood in stool, Pulse rate ****	0	1-2	3-4
	High level of Care (Yes, No)			
	Fever (Yes, >= 38C | No, < 38C)			
	Blood in Stool (Yes, No)			
	Pulse rate (High, >100 | Normal, <= 100)			
B	***Maximum num of stools per day***	1-3	4-6	>=7
C	***Duration of diarrhea***	1-2	3-4	>=5
D	***Stool Consistency***	1-2	3	4
E	***Presence of dehydration (thirsty, decreased urination, decreased skin turgor, dry mucus)****	1-2 (Some dehydration)	3 (Moderate dehydration)	>=4 (Severe dehydration)
F	***Behavioral signs (lethargy, Fatigue, Faint, Loss of work, Anorexia, e.t.c.) ****	1	2	3
	lost work, fatigue headache (0,1)			
	Bloating, Abdominal cramp, Anorexia, bloating, Flatulence (0,1)			
	Vomiting, Nausea (0, 1)			
* measured by taking the sum of the binarized parameters				

Supplementary Results

Multiplex PCR for detection of diarrheagenic pathogens.

Of the samples positive for DECs, enteropathogenic *E. coli* (EPEC; 49/217, 22.6%), enteroaggregative *E. coli* (EAEC; 45/217, 20.7%), and enterotoxigenic *E. coli* (ETEC; 42/217, 19.4%) were most commonly identified. Less frequently detected were diffusively adherent *E. coli* (DAEC; 31/217, 14.3%), Shiga toxin-producing *E. coli* (STEC; 20/217, 9.2%), and enteroinvasive *E.coli* (EIEC; 6/217, 2.8%). Consistent with the prior studies[1], we observed significantly higher detection rate of *Campylobacter* (OR: 13.9; 95% CI: 3.6-54.5; $P < 0.001$), ETEC (OR: 3.4; 95% CI: 1.7-6.9; $P < 0.001$), and norovirus (OR: 2.7; 95% CI: 1.1-6.4; $P = 0.03$) in diarrheal samples compared to non-diarrheal samples (Supplementary Table 13).

Antibiotic susceptibility testing of DEC isolates.

Rates of MDR were most common among DAEC (89.3%, 25/28), followed by EAEC (75.0%, 33/44), and EPEC (65.9%, 29/45) pathotypes, and less frequent among ETEC (59.5%, 22/37) and STEC (30.8%, 4/13) pathotypes. No resistance was detected against imipenem, ceftazidime, cefotaxime-clavulanate, nitrofurans, or amikacin. In contrast, high rates of resistance were detected against erythromycin (ERY; 95.9%, n=162), ampicillin (AMP; 65.7%, n=111), ticarcillin (TIC; 60.9%, n=103), tetracycline (TET; 58.0%, n=98), and trimethoprim-sulfamethoxazole (SXT; 53.3%, n=90) (Supplementary Table 14). Resistance to gentamycin (GEN; 3.6%, n=6), and 3rd and 4th generation cephalosporins (CRO; 1.8% n=3; FEP; 0.6%, n=1) was detected, but were relatively infrequent.

Isolate WGS and phylogeny.

The average genome size—representing both chromosomal and plasmid DNA—of the remaining 189 DEC isolates (non-diarrheal=139, diarrheal=50) was 5.1 Mb (range: 4.6-5.8 Mb) with a median N50 of 128 Kb. The multi-locus sequence type (MLST) profiles of these isolates represented 74 unique sequence types, with ST10 (42/189, 22.2%) and ST21 (15/189, 7.9%) being the most common in Clades A and B1. Among 189 sequenced DEC isolates, only ETEC (OR: 3.65; 95% CI: 1.5-8.7; $P = 0.002$) was significantly associated with diarrheal samples. No significant association was detected with other DEC types.

To further explore the diversity and distribution of AMR determinants harbored by isolates among travelers, we screened the draft genomes for known ARGs and point mutations that confer resistance (e.g., *gyrA, parC, parE*, and *pmrB*). In total, we identified 60 unique AMR determinants, including 21 core (present in ≥95% of isolates) and 39 accessory resistance determinants (Supplementary Table 15, see Methods). Consistent with previous findings, the core resistome of

DEC isolates included genes that mediate resistance to multiple drugs via efflux pumps[2]. The accessory resistome, however, was widely variable with no clear association with phylogroups or sample type (Supplementary Fig. 7a). The most common accessory ARGs were *emrE* efflux pumps (115 isolates, 60.9%), alleles of *sul* mediating resistance to sulfonamides (*sul1, sul2, sul3*, 88 isolates, 46.6%), ESBL-mediating bla_{TEM} ($bla_{TEM-148}$, $bla_{TEM-206}$, 82 isolates, 43.4%), trimethoprim resistance *dhfr* genes (76 isolates, 40.2%), and aminoglycoside resistance genes *aph6-Id* and *aph3"* (66 isolates, 34.9%). In addition, we detected point mutations in *gyrA* (S83L: 30, S83A: 14, D87N: 8, D87Y: 4, 48 isolates, 25.4%), *parC* (S80I: 9, A56T: 4, E84G: 2, E84V: 2, S57T: 2, 13 isolates, 6.9%), and *parE* (I355T: 3, I529L: 2, L416F: 2, S458A: 2, 9 isolates, 4.8%) quinolone resistance-determining region (QRDR) genes. The frequency of these prevalent accessory ARGs (present in ≥10% of isolates) was similar in DEC strains isolated from diarrheal and non-diarrheal samples (Supplementary Fig. 7b).

Supplementary Notes

Form –A Cohort Enrollment Form: Background information

First Name(s): ____
Surname(s): ____
Today's date (M/D/Y): _____/_____/_____ Age: _________ Blood Type _________ Sex: ( ) Male ( ) Female
E-mail address: ____
Mobile Phone: ____Native Language: ______________________________
Nationality: ____________________________________ Country of Residence: _________________________________
City of Residence _________________________________Occupation: ____

Date Arrived in Peru (M/D/Y) _____/_____/_____ Date arrived in Cusco (M/D/Y): _____/_____/_____
Planned date of departure (M/D/Y): _____/_____/_____
Lodging in Cusco:
( ) School Lodging Building __________________ Room Number ____________
( ) Host Family Address ____
( ) Other ____

Have you ever lived in a developing country greater than 1 month? ( ) Yes ( ) No
If so, which country or countries? ____

In the past 12 months, have you visited other countries prior to your arrival in Perú? ( ) Yes ( ) No
If so, please list the country / countries visited.

Country	Month/Year Visited	Number of Days Visited
________________________	_____/_____	_______________
________________________	_____/_____	_______________
________________________	_____/_____	_______________
________________________	_____/_____	_______________
________________________	_____/_____	_______________

Travel Medicine Information
Do you currently have health insurance with international coverage? ( ) Yes ( ) No
Did you receive any preventive medicine information before your trip? ( ) Yes ( ) No

What was the method of delivery for the travel medicine information?
( ) Paper handout or article ( ) A lecture ( ) Online training/website ( ) Clinic Visit
( ) Other ____

What topics were covered in this training (mark all that apply)? ( ) Malaria ( ) Diarrhea prevention ( ) Insect Protection ( ) Sun and environmental protection ( ) Altitude sickness ( ) Healthcare access ( ) Travel insurance ( ) Personal hygiene ( ) Food safety ( ) Pre-travel vaccinations ( ) Safe sex ( ) responsible alcohol consumption ( ) Country specific diseases ( ) Personal safety

Health Status
In the past two weeks, did you have diarrhea (3 or more loose or watery stools during any 24-hour period) or vomiting prior to your arrival in Cusco? ( ) Yes ( ) No
In the past 2 weeks have you taken antibiotics? ( ) Yes ( ) No
What antibiotics? ____

Vaccination Card Review:

Hepatitis A	( ) Yes	( ) No	( ) Not sure
Cholera	( ) Yes	( ) No	( ) Not sure
Typhoid (Oral)	( ) Yes	( ) No	( ) Not sure
Typhoid (Injectable)	( ) Yes	( ) No	( ) Not sure

Eating Habits
When eating in a developing country, do you eat raw vegetables?
( ) Always ( ) Almost always ( ) Sometimes ( ) Rarely ( ) Never

When eating in a developing country, do you eat raw fruits that have not been peeled?
( ) Always ( ) Almost always ( ) Sometimes ( ) Rarely ( ) Never

When traveling in a developing country, do you drink the local water?
( ) Always ( ) Almost always ( ) Sometimes ( ) Rarely ( ) Never

When traveling in a developing country, do you drink unbottled beverages or drinks with ice?
( ) Always ( ) Almost always ( ) Sometimes ( ) Rarely ( ) Never

Do you eat raw or undercooked meat or fish including ceviche?
( ) Always ( ) Almost always ( ) Sometimes ( ) Rarely ( ) Never

Do you add sauces to your food?
( ) Always ( ) Almost always ( ) Sometimes ( ) Rarely ( ) Never

Do you wash your hands before eating?
( ) Always ( ) Almost always ( ) Sometimes ( ) Rarely ( ) Never

Medical History (to be filled out by the physician)
Allergies: ____

Pertinent Medical History:
( ) GERD ( ) PUD ( ) Crohn's Disease ( ) Diarrhea Predominant IBS
( ) Dyspepsia ( ) HIV ( ) Ulcerative Colitis ( ) Constipation Predominant IBS
( ) Lactose Intolerant ( ) Celiac Disease ( ) Other, list below
____
____
____
____
____

Are you currently taking any antibiotics….
chronically for an illness? ( ) Yes ( ) No
for prophylaxis? ( ) Yes ( ) No
Name of antibiotic(s):______________________________

Current chronic medications:
____
____
____
____
____
____
____
____
____

Medications brought with you:
( ) ciprofloxacin ( ) azithromycin ( ) rifaxamin
( ) levofloxacin ( ) Bactrim/Septra ( ) doxycycline
( ) amoxicillin ( ) Augmentin ( ) probiotics
( ) Pepto-Bismol ( ) Imodium ( ) Lomotil
( ) Buscopan ( ) Phenergan ( ) steroids
( ) acetazolamide ( ) acetaminophen ( ) NSAIDS
( ) antacids ( ) PPI ( ) H_2 blocker
( ) Benadryl ( ) meclizine ( ) scopolamine
( ) other ____

How interested would you be in receiving a vaccine that would protect against traveler's diarrhea?
( ) not interested ( ) somewhat interested ( ) interested ( ) very interested

How willing would you be in participating in a future clinical vaccine trial toward preventing diarrhea?
( ) not interested ( ) somewhat interested ( ) interested ( ) very interested

To be filled out by study site staff:
Notes:____
____
____
____

Form –B GI Illness Symptoms

GI Illness Episode Form Participant Code ______________

First Name(s) ________________________ **Today's Date** ____/____/____

Surname(s) ________________________

Date/Time of Onset of Illness Time (24-hour format) _________ Date ____/____/____ **Date Well** ____/____/____

Days from start of illness

Symptoms	1	2	3	4	5	6	7	8	9	10	11	12	13	14
Interviewer's Initials														
Date Year: 20__ __	/	/	/	/	/	/	/	/	/	/	/	/	/	/
Days on Antibiotics														
Stools Last 24hrs														
Stool Consistency														
Stool Quality														
Lost Work/Decreased Activity														
Loss of Bowel Control/Urgency														
Blood in Stools														
Vomiting last 24hrs														
Nausea														
Anorexia														
Flatulence														
Bloating														
Adominal Cramping														
Tenesmus (Straining)														
Subjective Fever														
Headaches														
Muscle Cramps														
Rash														
Joint Pain														
Thirsty														
Descreased Urination														
Dark Urine Color														
Feels Faint/Dizzy Upon Standing														
Fatigue														

Last Name(s) ______________________________ Participant Code ________________

Days from start of illness

Signs	1	2	3	4	5	6	7	8	9	10	11	12	13	14
Date Year: 20__ __	/	/	/	/	/	/	/	/	/	/	/	/	/	/
Temperature														
Pulse Laying*														
Pulse Sitting														
Pulse Standing*														
Orthostatic Tachycardia*														
Blood Pressure Laying*														
Blood Pressure Sitting														
Blood Pressure Standing*														
Orthostatic Hypotension*														
Lethargy														
Decreased Skin Turgor														
Dry Mucus Membranes														

**Tilts only have to be performed during the initial evaluation or if the patient reports orthostatic symptoms.*

Have you had prior medical attention for this diarrhea? ( ) Yes ( ) No

If so, where did you receive medical attention: ____________________________ Date seen: ____/____/____

Have you been self-medicating? ( ) Yes ( ) No If so, what medication(s):________________________

Did you get the medication from: ( ) your personal stash ( ) a friend ( ) a pharmacy in Perú ( ) new prescription

Do you know anyone else who was recently sick with similar symptoms? ( ) Yes ( ) No

If so, was it a: ( ) fellow class mate ( ) local person ( ) new visitor ( ) school staff ( ) girl/boy friend ( ) Other

Initial Treatment: **Date/Time of Treatment:** **Time (24-hour format)** _________ **Date** ____/____/____

( ) Increase Fluid Intake ( ) ORS Solution ( ) Phenergan ( )IV fluids (Type ___________ Qty:_______L)

( ) Immodium ( ) Pepto-Bismol ( ) Tylenol ( )Other:_______________________________

Name/dose/frequency

( ) Antibiotic_______________________________________

Name/dose/frequency

Was patient refered to a higher level of car( ) Yes ( ) No **Stool Collected and Processed:** ( ) Yes ( ) No

Doctor's Notes

Supplementary References

1 Bodhidatta, L. *et al.* Epidemiology and etiology of Traveler's diarrhea in Bangkok, Thailand, a case-control study. *Tropical diseases, travel medicine and vaccines* **5**, 9, doi:10.1186/s40794-019-0085-9 (2019).

2 Goldstone, R. J. & Smith, D. G. E. A population genomics approach to exploiting the accessory 'resistome' of Escherichia coli. *Microbial genomics* **3**, e000108, doi:10.1099/mgen.0.000108 (2017).
