# Supplementary material for: Impact of international travel and diarrhea on gut microbiome and resistome dynamics
Source: Nat Commun. 2022 Dec 5;13:7485. doi: 10.1038/s41467-022-34862-w (PMC9722912; doi:10.1038/s41467-022-34862-w)
Supplement: Supplementary file 3 — Description of Additional Supplementary Files [file 41467_2022_34862_MOESM3_ESM.docx]

**Description of Additional Supplementary Files**

Filename: Supplementary Data 1

Description: Antibiotic selections of 21 metagenomics pooled libraries constructed from 210 travelers gut microbiome

Filename: Supplementary Data 2

Description: Metadata of ShortBRED markers used for identification and quantification of AR genes in metagenomics samples
