# Supplementary material for: Impact of international travel and diarrhea on gut microbiome and resistome dynamics
Source: Nat Commun. 2022 Dec 5;13:7485. doi: 10.1038/s41467-022-34862-w (PMC9722912; doi:10.1038/s41467-022-34862-w)
Supplement: Supplementary file 6 — Reporting Summary [file 41467_2022_34862_MOESM6_ESM.pdf]

nature portfolio

Corresponding author(s): Manish Boolchandani, Gautam Dantas, Mark P. Simons

Last updated by author(s): Oct 15, 2022

Reporting Summary

Nature Portfolio wishes to improve the reproducibility of the work that we publish. This form provides structure for consistency and transparency in reporting. For further information on Nature Portfolio policies, see our Editorial Policies and the Editorial Policy Checklist.

Statistics

For all statistical analyses, confirm that the following items are present in the figure legend, table legend, main text, or Methods section.

n/a	Confirmed	
☐	☒	The exact sample size (n) for each experimental group/condition, given as a discrete number and unit of measurement
☐	☒	A statement on whether measurements were taken from distinct samples or whether the same sample was measured repeatedly
☐	☒	The statistical test(s) used AND whether they are one- or two-sided<br>*Only common tests should be described solely by name; describe more complex techniques in the Methods section.*
☐	☒	A description of all covariates tested
☐	☒	A description of any assumptions or corrections, such as tests of normality and adjustment for multiple comparisons
☐	☒	A full description of the statistical parameters including central tendency (e.g. means) or other basic estimates (e.g. regression coefficient) AND variation (e.g. standard deviation) or associated estimates of uncertainty (e.g. confidence intervals)
☐	☒	For null hypothesis testing, the test statistic (e.g. F, t, r) with confidence intervals, effect sizes, degrees of freedom and P value noted<br>*Give P values as exact values whenever suitable.*
☒	☐	For Bayesian analysis, information on the choice of priors and Markov chain Monte Carlo settings
☒	☐	For hierarchical and complex designs, identification of the appropriate level for tests and full reporting of outcomes
☒	☐	Estimates of effect sizes (e.g. Cohen's d, Pearson's r), indicating how they were calculated

Our web collection on statistics for biologists contains articles on many of the points above.

Software and code

Policy information about availability of computer code

Data collection

No specific software was used for data collection

Data analysis

All of the applied software is cited in the Methods section. We list the tools here:
- Trimmomatic v0.36: quality filtering and adapter trimming
- DeconSeq v0.4.3: remove contaminating human DNA
- MetaPhlAn 2.0: metagenome taxonomic profiling
- HUMAnN2 v0.9.4: metagenome functional profiling
- ShortBRED v0.9.4: metagenome resistome profiling
- PARFuMS v1.1.0: assembly of functionally selected fragments
- MetaGeneMark v3.2.6: gene prediction
- SPAdes v3.11.0: genome assembly
- CheckM v1.0.13: isolates assembly quality assessment
- Prokka v1.12: isolates genome annotation
- Roary v3.8.0: pangenome analysis of isolates
- RAXML v8.2.11: maximum likelihood phylogeny construction
- Resfams v1.2: resistance gene annotation in functional selections
- Resfinder v4.0: annotation of antibiotics resistance genes
- CARD v3.0.5: annotation of antibiotic resistance genes
- NCBI-AMRFinderPlus v3.0.12: annotation of antibitioc resistance genes
- Virulencefinder v2.0.4: annotation virulence factors in isolate genomes
- VFDB: virulence factor database (downloaded on 06-04-2020)
- MGEfinder v1.0.2: annotation of mobile genetic elements in isolate genomes

	- Serotypefinder v2.0.1: identification of serotype in E.coli genomes<br>- MLST v2.11: multiloucs sequence typing of isolate genomes<br>- Strainsifter v1.0: comparison of phylogenetic relatedness of isolates<br>- SparCC v0.1.0: co-occurrence network analysis<br>- R v3.6.3 for statistical analysis

For manuscripts utilizing custom algorithms or software that are central to the research but not yet described in published literature, software must be made available to editors and reviewers. We strongly encourage code deposition in a community repository (e.g. GitHub). See the Nature Portfolio guidelines for submitting code & software for further information.

Data

Policy information about availability of data

All manuscripts must include a data availability statement. This statement should provide the following information, where applicable:

- Accession codes, unique identifiers, or web links for publicly available datasets
- A description of any restrictions on data availability
- For clinical datasets or third party data, please ensure that the statement adheres to our policy

All data from shotgun metagenomics, isolates and functional metagenomics sequencing are available from the NCBI SRA under BioProject ID PRJNA698223

Field-specific reporting

Please select the one below that is the best fit for your research. If you are not sure, read the appropriate sections before making your selection.

☒ Life sciences ☐ Behavioural & social sciences ☐ Ecological, evolutionary & environmental sciences

For a reference copy of the document with all sections, see nature.com/documents/nr-reporting-summary-flat.pdf

Life sciences study design

All studies must disclose on these points even when the disclosure is negative.

Sample size	No sample size calcluations were performed because this was a non-interventional, observational study that leveraged existing samples for which recruitment, enrollment, and sample collection had concluded and anticipated effect sizes were unclear.
Data exclusions	Low quality bases were trimmed from reads prior to analysis to prevent miscalling.<br>Genes with annotations “Efflux Regulator”, “Operon Regulator”, and “Other” were removed from analysis consideration due to their lower specificity to antimicrobial resistance compared to other genes in the database.
Replication	- To confirm the E.coli pathotypes, both Multiplex PCR and whole genome sequencing of diarrheagenic E.coli were performed.<br>- Metagenomic DNA from multiple individuals (n=10) were pooled during functional metagenomics to reduce random variation.
Randomization	This work is a non-interventional observational study, so randomization of participants was not necessary.
Blinding	This work is a non-interventional observational study and investigators had no direct contact with participants recruitment, so blinding is not relevant.

Reporting for specific materials, systems and methods

We require information from authors about some types of materials, experimental systems and methods used in many studies. Here, indicate whether each material, system or method listed is relevant to your study. If you are not sure if a list item applies to your research, read the appropriate section before selecting a response.

Materials & experimental systems

n/a	Involved in the study
☒	☐ Antibodies
☒	☐ Eukaryotic cell lines
☒	☐ Palaeontology and archaeology
☒	☐ Animals and other organisms
☐	☒ Human research participants
☒	☐ Clinical data
☒	☐ Dual use research of concern

Methods

n/a	Involved in the study
☒	☐ ChIP-seq
☒	☐ Flow cytometry
☒	☐ MRI-based neuroimaging

Human research participants

Policy information about studies involving human research participants

Population characteristics	The characteristics of the study population are detailed in Supplementary Table 1.
Recruitment	International travelers aged ≥18 years, able to understand written English, and enrolled at the school as students were eligible. The study physician explained the purpose and procedures of the study to potential participants within 24 to 48 hours of their arrival in Cusco city. There was no selection-bias during an enrollment process. Subjects who agreed to participate and signed an informed consent form were asked to complete a standardized enrollment questionnaire that include baseline demographic, epidemiologic data, medical, and travel history data. Subjects were also provided a stool collection kit and instructions to collect a baseline stool sample with their next bowel movement. Weekly stool samples were collected from subjects throughout their stay, and a weekly questionnaire collected data on unreported diarrheal episodes and dietary habits. Subjects were instructed to immediately communicate with the study physician when having a diarrheal episode and before taking any medication (e.g., antibiotics). Subjects reporting diarrhea were asked to provide an extra stool sample and were examined daily. Physical exam findings, symptoms, duration, and medications were documented. Samples were classified as non-diarrhea (ND) or diarrhea (D) based on whether they were collected during a diarrheal episode or not. Samples were also given a stool grade based on the Bristol stool form scale (BSS) of stool consistency, ranging from grade 1 (normal, hard) to grade 4 (loose, diarrheal).
Ethics oversight	The study protocol and its amendments were reviewed and approved by the Institutional Ethics Committee of the Universidad Peruana Cayateno Heredia, the Institutional Review Board of the US Naval Medical Research Unit No. 6 (NAMRU-6), and the Washington University in St. Louis Institutional Review Board.

Note that full information on the approval of the study protocol must also be provided in the manuscript.
